# pH plays a role in the mode of action of trimethoprim on *Escherichia coli*

**DOI:** 10.1371/journal.pone.0200272

**Published:** 2018-07-13

**Authors:** Haitham AlRabiah, J. William Allwood, Elon Correa, Yun Xu, Royston Goodacre

**Affiliations:** 1 School of Chemistry and Manchester Institute of Biotechnology, University of Manchester, Manchester, United Kingdom; 2 Department of Pharmaceutical Chemistry, College of Pharmacy, King Saud University, Riyadh, Saudi Arabia; 3 Environmental and Biochemical Sciences Group, The James Hutton Institute, Invergowrie, Dundee, Scotland United Kingdom; Universite Paris Diderot, FRANCE

## Abstract

Metabolomics-based approaches were applied to understand interactions of trimethoprim with *Escherichia coli* K-12 at sub-minimum inhibitory concentrations (MIC≈0.2, 0.03 and 0.003 mg L^-1^). Trimethoprim inhibits dihydrofolate reductase and thereby is an indirect inhibitor of nucleic acid synthesis. Due to the basicity of trimethoprim, two pH levels (5 and 7) were selected which mimicked healthy urine pH. This also allowed investigation of the effect on bacterial metabolism when trimethoprim exists in different ionization states. UHPLC-MS was employed to detect trimethoprim molecules inside the bacterial cell and this showed that at pH 7 more of the drug was recovered compared to pH 5; this correlated with classical growth curve measurements. FT-IR spectroscopy was used to establish recovery of reproducible phenotypes under all 8 conditions (3 drug levels and control in 2 pH levels) and GC-MS was used to generate global metabolic profiles. In addition to finding direct mode-of-action effects where nucleotides were decreased at pH 7 with increasing trimethoprim levels, off-target pH-related effects were observed for many amino acids. Additionally, stress-related effects were observed where the osmoprotectant trehalose was higher at increased antibiotic levels at pH 7. This correlated with glucose and fructose consumption and increase in pyruvate-related products as well as lactate and alanine. Alanine is a known regulator of sugar metabolism and this increase may be to enhance sugar consumption and thus trehalose production. These results provide a wider view of the action of trimethoprim. Metabolomics indicated alternative metabolism areas to be investigated to further understand the off-target effects of trimethoprim.

## Introduction

One of the most effective mechanisms of drug action is enzyme inhibition, although often the mechanisms underlying the specific modes of action are not always fully understood [1,2]. This is typically because there is often an assumption that an antibiotic is an inhibitor of a specific enzyme (or indeed another target), not realizing that this chemical can have other ‘off-target’ effects, such as binding to unidentified enzymes or indirect interactions with other metabolic pathways that may affect the performance of the drug [3]. The range and complexity of cellular chemical reactions (the metabolic network) increase the challenge of understanding the mode of action of antibiotics as multiple changes in the metabolic network occur during antibiotic-induced abiotic perturbation [3]. It is believed that metabolomics is a powerful approach that can be used to measure phenotypic response following antibiotic challenge [4]. Analysis of metabolomes has increased dramatically in recent years due to the introduction of ultra-high resolution mass spectrometers [5] which allow accurate identification of small molecules in complex extracts [6,7]. Indeed, this approach has already been used for analyzing various pathogen phenotypes [4].

The biosynthesis of essential metabolites, such as purines, thymine, glycine and methionine, generally uses folates as cofactors that either add or subtract one-carbon units. Several therapeutics including anticancer agents, like pemetrexed, and antibiotics, such as trimethoprim, target folate metabolism [8,9]. Folates can be found in three different oxidation/reduction states (*viz*., dihydrofolate (DHF), folate or pteroylglutamate and tetrahydrofolate (THF)) and are synthesized from guanosine 5'-triphosphate (GTP), *p*-aminobenzoic acid (*p*ABA) and glutamates. The dihydrofolate reductase (DHFR) enzyme reduces DHF to THF using NADPH as the electron donor. Downstream to this various folates such as 5-methyl-THF, 5-formyl-THF, 5-formimino-THF, 10-formyl-THF, 5,10-methenyl-THF and 5,10-methylene-THF can be formed by substituting tetrahydrofolate species with one-carbon units to produce active donors involved in various biosynthetic reactions [3].

Urinary tract infections (UTIs) are very common; it is estimated that during the female lifespan 50% are likely to acquire a UTI [10,11]. A study of midstream urine samples spanning 252 centres in 17 countries revealed that *Escherichia coli* accounted for 77% of all isolates, 80% of general infections and 40% of nosocomial infections [12,13]. The weak base antifolate drug trimethoprim resulted from the work of Hitchings and his group across the 1940-60s at Burroughs Wellcome, USA. Hitchings and colleagues studied the cellular actions of biologically important heterocyclic purines and pyrimidines on the basis that interference in associated processes might lead to the discovery of therapeutic effects [14]. The Hitchings group successfully developed several therapeutic active agents and Hitchings and Elion were part awarded the Nobel Prize for Physiology and Medicine in 1988 for the discovery of important principles in drug treatment [15]. Trimethoprim is still used therapeutically today and is particularly effective in treating both community and nosocomial UTIs [16]. Trimethoprim is mainly used to treat uncomplicated UTIs and acts by inhibiting bacterial DHFR, reducing active tetrahydrofolates which are needed for synthesis of various essential metabolites and these are important precursors for nucleic acid biosynthesis [17].

In this study, *E*. *coli* K-12 was challenged with different concentrations of trimethoprim at different pH levels (pH 5 and 7) and analyzed by Fourier transform infrared (FT-IR) spectroscopy and gas chromatography-mass spectrometry (GC-MS) to produce global snapshots of the bacterial phenotypic and untargeted metabolic profiles, respectively. We believe this metabolomics-based approach provides a greater level of insight and understanding of trimethoprim’s mode(s) of action. The reason for including varying pH in this investigation is because trimethoprim is largely excreted unchanged in human urine and in a healthy person the normal pH range of urine is between 4.6 and 7.5 [14,18]. The bacterial intracellular trimethoprim levels were estimated using liquid chromatography-mass spectrometry (LC-MS) as this antibiotic is ionized within this pH range and this may affect its ability to be transported across the cell membrane [19].

## Results

For the experiments conducted in this study we chose to use *E*. *coli* K-12 strain MG1655 as the full genome sequence of this microorganism is available which has allowed construction of the metabolic pathways in this bacterium. The latter is useful as this allows one to use KEGG for metabolite pathway analysis (*vide infra*). A potential limitation of our study is that we have not used wild-type pathogenic *E*. *coli* strains. As reported in [20] *E*. *coli* K-12 is a laboratory strain that has become adapted to life outside of the host and such adaptation may mean that this strain has lost its ability to survive in a human environment.

### Determination of optimum growth conditions

**Fig 1:**
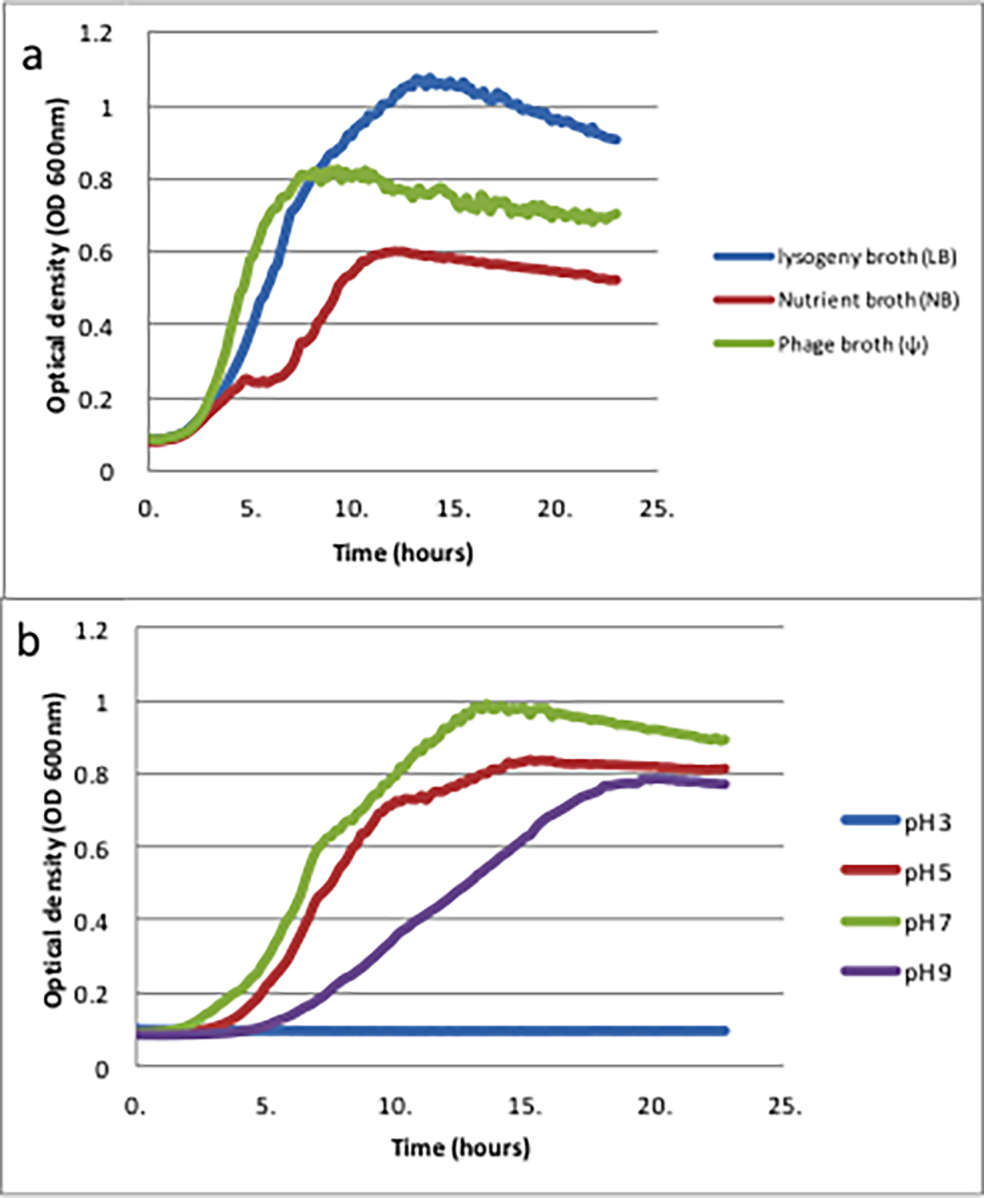
Growth curves of *E*. *coli* K-12. (a) Growth curves of *E*. *coli* K-12 in three different media. Media: the blue plot indicates LB; red, NB and green, Ψ. (b) Growth curves of E. coli K-12 at four different pH values in the same LB medium. The blue plot indicates pH 3; red pH5; green pH 7 and purple pH 9. Six replicate growth curves were conducted and a typical growth curve for each condition is shown; the other five growth curves showed similar dynamics.

*E*. *coli* K-12 was exposed to different concentrations of trimethoprim at different pH levels, and preliminary experiments established that the optimum medium to use was LB (Fig 1A), which was therefore used throughout this work. *E*. *coli* was cultured in different pH environments: 3, 5, 7 or 9. No growth occurred in extreme acidic conditions (Fig 1B), perhaps because when pH<4, this environment typically has a bactericidal effect on *E*. *coli* [21–23]. The reason we consider the effect of pH on bacterial growth, and subsequently investigate the effect of trimethoprim challenge on *E*. *coli* at carrying pH, was to have the bacteria and antibiotic in an environment that mimics the pH of natural urine environment which affects drug ionization; pKa of trimethoprim ≈7.4 [24] and the ionization of the NH_2_ groups is discussed later.

The pH of the cytoplasm (pH_i_) of *E*. *coli* is regulated between 7.2 and 7.8 [25]. If changes occur in the environmental pH (pH_o_), the bacterium tries to preserve nucleic acid and protein stability, as well as enzymatic activity, by maintaining this range [25]. *E*. *coli* uses several mechanisms to maintain pH homeostasis and one of the most common appears to be cation-dependant proton flux [26]. From Fig 1B, when pH = 7, which results in ΔpH (pH_i_ ─ pH_o_) of approximately zero, the highest growth occurs. Therefore, pH 7 is the optimum of the three pH levels.

Although *E*. *coli* can preserve the activity of its nucleic acids, proteins and enzymes in a pH range from 4.5 to 9 [25], a comparison between pH 5 and pH 9 showed that at pH 5 the growth curve was higher (Fig 1B) indicating that *E*. *coli* K-12 can adapt to mildly acidic conditions better than basic conditions maybe because under alkaline conditions of pH 9, homeostasis makes high energy demands on the cell and protons are lost [27]. In addition, when *E*. *coli* is cultured in a medium that contains amino acids, e.g. LB, it has a greater possibility of surviving in acidic conditions [28].

A microscopic view of *E*. *coli* at different pH (S1 Fig) reveals subtle variations in cell size. At pH 5 and 7, the cells are typical of *E*. *coli*, whilst at pH 9, cells are slightly shorter and are affected by this mildly alkaline environment; these are estimated by EM (S1 Fig) to be *ca*. 2.0 μm in length compared to *ca*. 2.5 μm.

### The MIC of trimethoprim in *E*. *coli* K-12

In order to measure subtle antibiotic effects on *E*. *coli*, it is important to use levels that are below the MIC of trimethoprim, else all that will be measured is cell death and hence biomass level differences rather than metabolic shifts. Therefore, *E*. *coli* was challenged with different concentrations of trimethoprim. From S2 Fig, it can be seen that the MIC of trimethoprim in *E*. *coli* K-12 under optimum conditions (LB medium at pH 7) is approximately 0.2 mg L^-1^, therefore this and lower concentrations (0.03 and 0.003 mg L^-1^) were chosen to challenge *E*. *coli* K-12 in order to determine the effect of the antibiotic at a range of concentrations from the MIC to levels that have little or no effect on growth.

### Challenge of *E*. *coli* K-12 under different pH and antibiotic conditions

Trimethoprim is a heterocyclic weak base with pKa 7.4 [24] (S3A Fig). It acts on dihydrofolate reductase thus inhibiting nucleic acid synthesis. The effect of different pH levels on the drug molecules can be characterized according to the Henderson-Hasselbalch equation for weak bases which can be rewritten in a simple way (Eq 1) to calculate the percentage of ionization [29]:

$$\%\;ionization=\frac{10^{pKa-pH}}{1+10^{pKa-pH}}\times 100$$ (1)

S3 Fig a shows that at pH 9 there was no growth when bacteria were exposed to 0.2 mg L^-1^ of the antibiotic. Ionization calculations indicate that at this pH, trimethoprim remained largely non-ionized (only 2.5% ionized), which facilitated its penetration through the cell membrane of the microbial cell [30], thus inhibiting growth. At pH 7, which is the optimum pH for growth of the bacterium, it was found that 71.5% of the drug was ionized; the non-ionized remainder was able to penetrate and had a measurable effect (S3C Fig).

By contrast, at pH 5, 99.6% of trimethoprim was ionized, which reduced its ability to penetrate the cell membrane. Although at 0.2 mg L^-1^ there was a slight effect on bacterial growth (S3D Fig), indicating that trimethoprim passed into the cell, at lower dose levels there was no clear effect on growth. This may be due to the ability of trimethoprim (molecular weight 290.3) to pass through porins, which are transmembrane proteins in the outer membrane that hydrophilic molecules (molecular weight up to 600 in the case of *E*. *coli*) can penetrate by passive diffusion [31].

In order to establish whether trimethoprim penetrates the bacterial cell wall, targeted LC-MS was conducted to quantify the drug within *E*. *coli*. This work focused on pH 5 for the arguments made above and this was compared with pH 7 as a control, and of course both of these pH levels are relevant as they are within the normal pH range of human urine.

**Fig 2:**
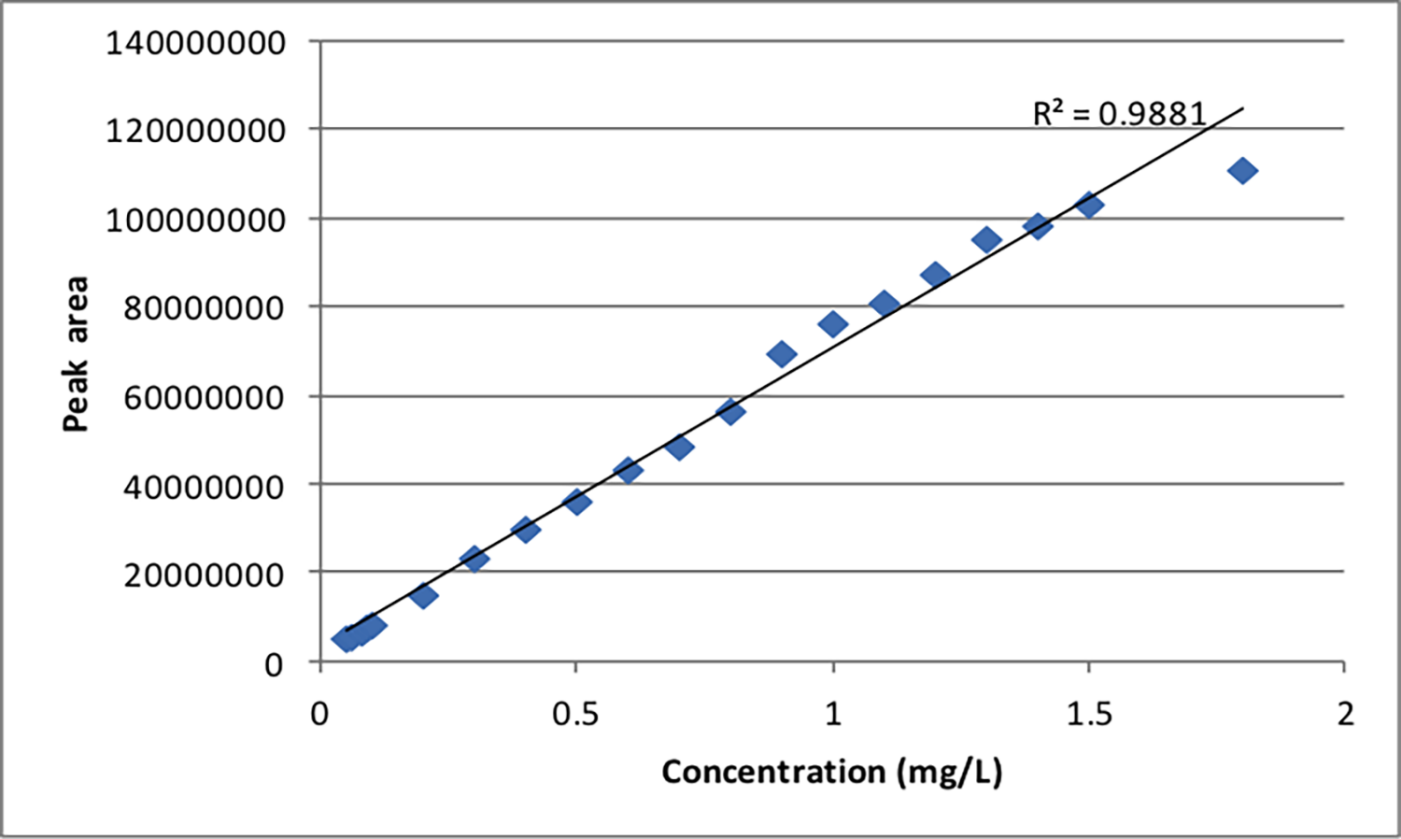
Calibration curve for LC-MS. The curve was built from 20 different gradient concentrations of trimethoprim; see Supporting S1 Text for information on the concentrations of trimethoprim used to construct the standard curve.

**Fig 3:**
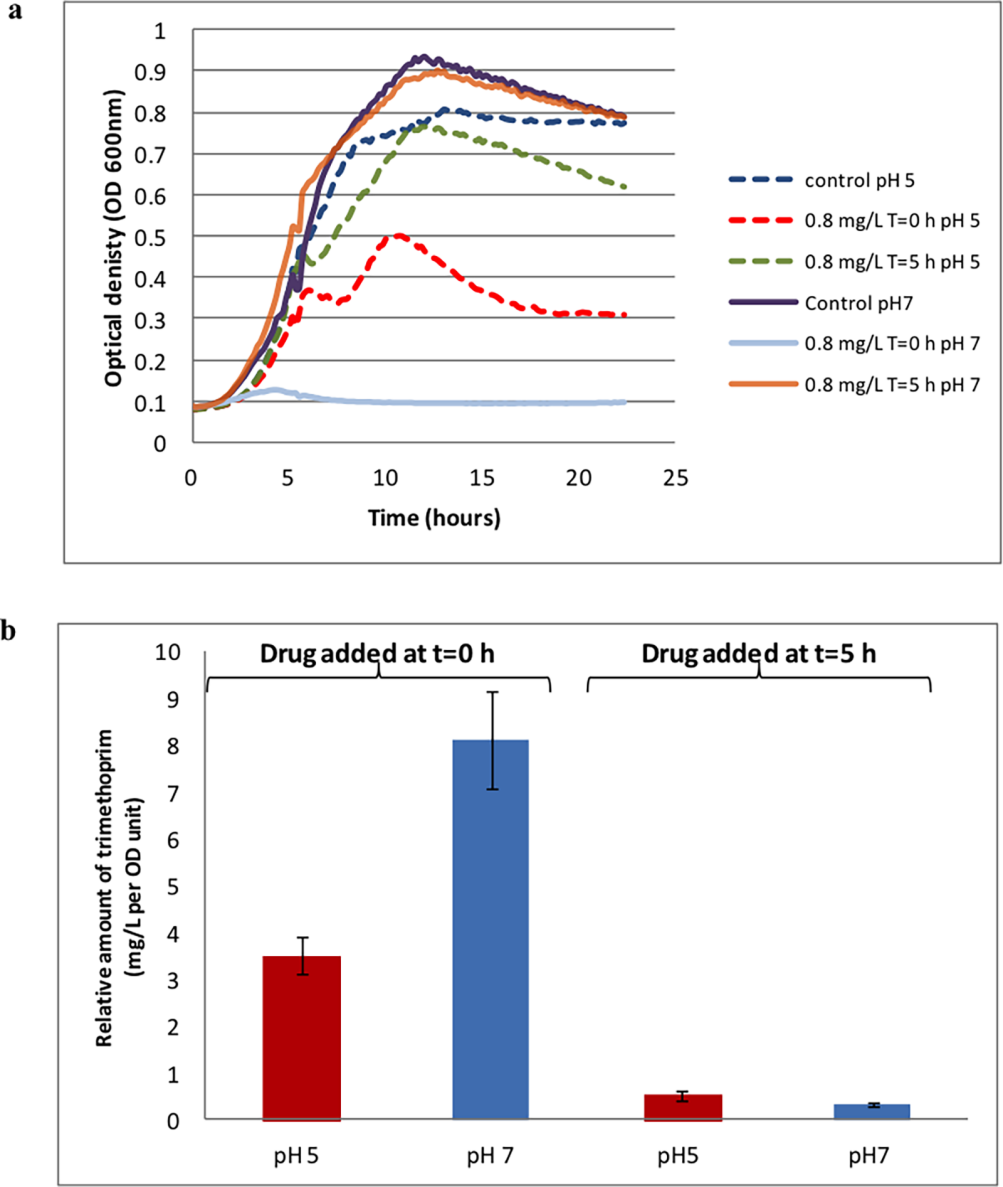
Growth characteristics of *E*. *coli*. (a) Growth curves of *E*. *coli* K-12 at pH 5 (dashed line) and pH 7 (solid line). For pH 5, the dashed blue line represents control samples, dashed red indicates samples challenged with 0.8 mg L^-1^ of trimethoprim added at the beginning of the lag phase (t = 0 h) and dashed green denotes samples challenged with 0.8 mg L^-1^ of trimethoprim and added at mid-exponential phase (t = 5 h). For pH 7, the solid purple line represents control samples, solid light blue indicates samples challenged with 0.8 mg L^-1^ of trimethoprim added at the beginning of the lag phase (t = 0 h) and solid orange denotes samples challenged with 0.8 mg L^-1^ of trimethoprim and added at the exponential phase (t = 5 h). (b) Column chart representing relative *E*. *coli* intracellular levels of trimethoprim after challenging with 0.8 mg L^-1^ of the drug at pH 5 (red columns) and pH 7 (blue columns) at different growth stages (time = 0 and 5 h) as detected by LC-MS analysis after cells were grown for a total of 18 h. Six replicate growth curves were conducted and a typical growth curve for each condition is shown; the other five growth curves showed similar dynamics.

Generation of a standard curve for trimethoprim (Fig 2) established that at a level of 0.2 mg L^-1^ the detectable signal with LC-MS was poor. Therefore, 0.8 mg L^-1^ of trimethoprim was used to ensure that the drug could be detected by LC-MS. The effect on growth of *E*. *coli* is shown in Fig 3A and this shows that the drug had the strongest effect when added at the beginning of the culture (lag phase) at pH 7 (light blue curve) than at pH 5 (red curve). These curves agree with the data presented in S3C and S3D Fig (i.e. in terms of the drug effect at 0.2 mg L^-1^) and the literature which shows that trimethoprim has a profound effect during bacterial lag phase [32]. By contrast, when the drug was added after 5 h (during the exponential phase) there was no effect at pH 7 (orange curve) and only a slight effect on the growth curve at pH 5 (green curve) compared with control. This means that the integrity of bacteria is not compromised and biomass yield is high enough to allow accurate estimations of drug uptake, or otherwise, from these bacteria.

As detailed in Supporting Information (S1 Text), LC-MS was used to estimate the relative quantification of trimethoprim inside the cell (Fig 3B). As expected, the highest level of the drug was recovered from cells at pH 7 when trimethoprim was added at the beginning of the lag phase (t = 0 h), while the second highest was when the bacterium was challenged at t = 0 h with the drug at pH 5. This relative difference is due to the ionization of trimethoprim where the NH_2_ groups are ionized to NH_3_^+^ and thus the nearly fully ionized drug is presumed to not be able to enter the cell via porins.

It was interesting to observe that when the bacterium was challenged at mid-exponential phase (t = 5 h) at both pH 5 and 7, regardless of the ionized state of the drug, the intracellular levels of the antibiotic (Fig 3B) were at their lowest, and this is presumably why these cultures exhibited little reduction in growth rate (Fig 3A).

### Metabolic fingerprinting of *E*. *coli* K-12 with FT-IR

*E*. *coli* K-12 cells were cultured in LB medium at pH 5, 7 and 9, and three concentrations of trimethoprim (0.003, 0.03 and 0.2 mg L^-1^), giving 12 different conditions including three controls. All cultures were repeated six times (six biological replicate) and each of these were analyse in triplicate (technical replicates). FT-IR spectra were recorded from the dried cell biomass in transmission mode at all three pH levels. From S4 Fig at pH 9 some spectra gave the response of empty wells (flat baselines), resulting from the complete inhibition of *E*. *coli* growth at this pH; these corresponded to exposure to MIC levels (0.2 mg L^-1^) of trimethoprim. Due to the very low (or in some case no) signal, all FT-IR data from cultures at pH 9 were excluded from the remaining experiments. Prior to multivariate analysis, appropriate scaling and normalization was conducted for all 8 conditions at pH 5 and 7; the effects of these mathematical operations are shown in S5 Fig. Subsequently, principal components analysis (PCA) and supervised principal components analysis-discriminant function analysis (PC-DFA) were applied to these spectra.

**Fig 4:**
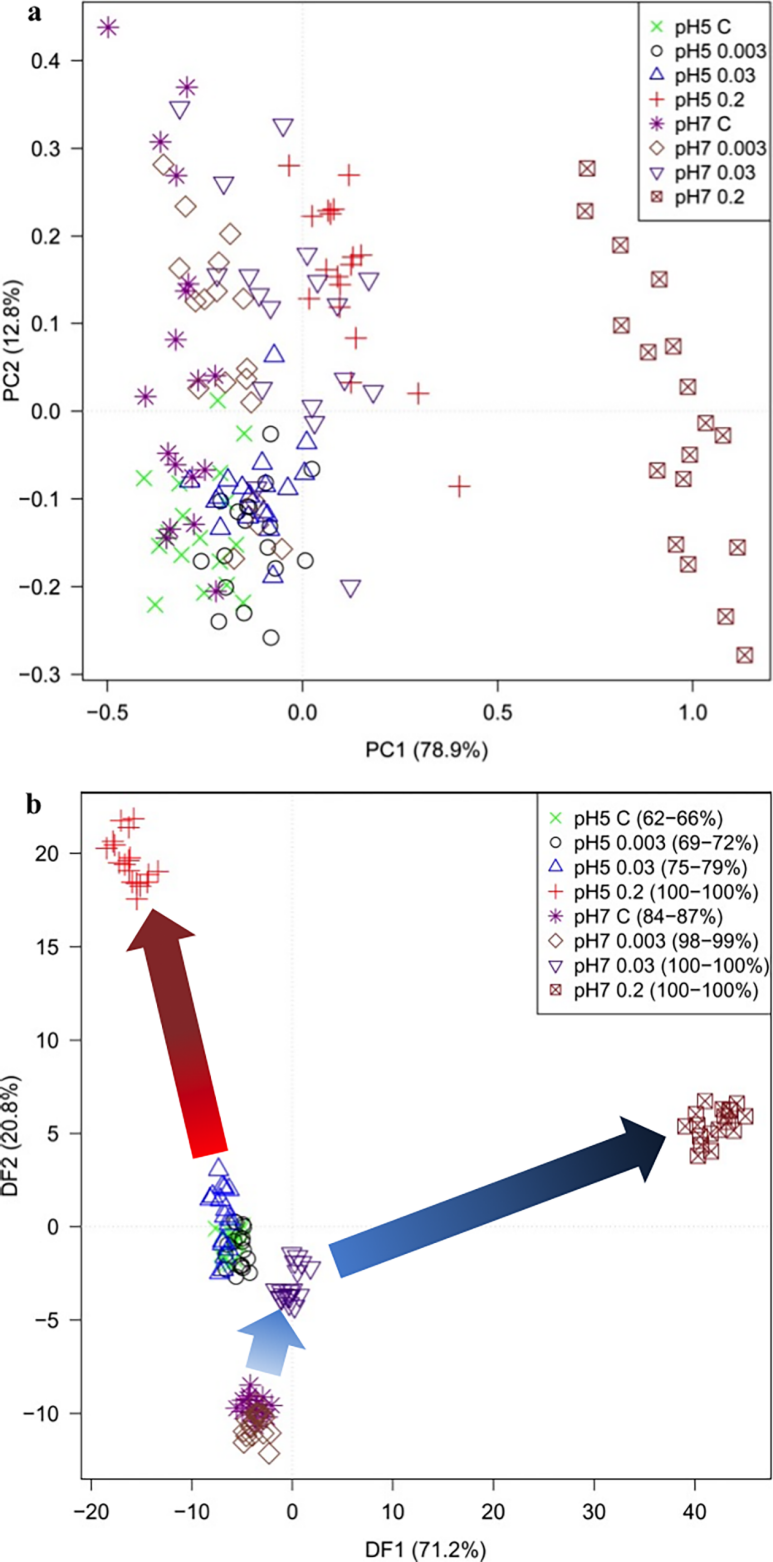
PCA and DFA on FT-IR spectra reveal pH and trimethoprim effects. (a) PCA scores plot of PC1 *vs*. PC2 after CO_2_ removal around 2350 cm^-1^ and EMSC scaling. The total explained variance (TEV) of PC1 is 78.9% and for PC2 is 12.8%. (b) PC-DFA score plots of pH 5 and 7 samples. 20 PCs were extracted from PCA and used as inputs to DFA. These 20 PCs explain 99% of TEV; the legend in the plot shows the 95% confidence interval (CI) for the correct classification of the eight conditions. C, control.

Fig 4A shows the PCA scores plot of PC1 *versus* PC2; the variance explained by PC1 is 78.9% and by PC2 12.8%. It can be seen that the largest difference in these samples is the dominant phenotypic shift in *E*. *coli* due to exposure to 0.2 mg L^-1^ of trimethoprim at pH 7 which are clearly separated from all other samples in PC1. Next, PC-DFA was applied and this was based upon the first 20 PCs (accounting for a total explained variance (TEV) of 99.99%) and the *a priori* knowledge of the different conditions (8 classes in total), and was validated as detailed above (the 95% confidence ranges are provided in parentheses for the 8 groups in Fig 4B). It is clear from this PC-DFA score plot that cells exposed to 0.2 mg L^-1^ at pH 5 could now also be clearly differentiated. Moreover, PC-DF1 which accounts for the most group variance allows separation from all the cells exposed to pH 7, which are located on the right hand side for this plot, while pH 5 are found on the left hand side. In addition, PC-DF2 generally explains the exposure of cells in both pH environments to increasing levels of trimethoprim.

**Fig 5:**
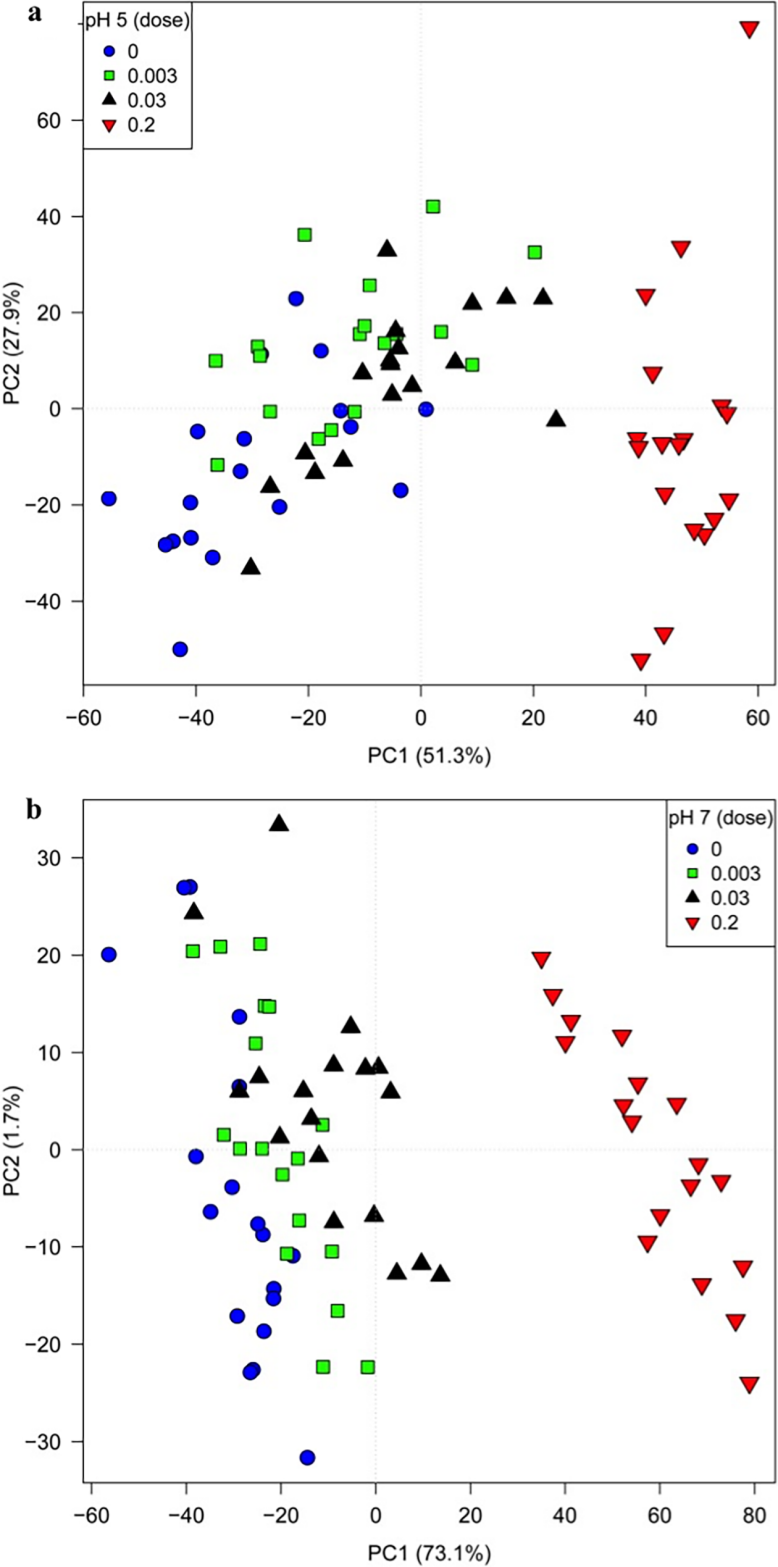
Multi-block PCA score plots from FT-IR spectra. The plot shows the relationship between the effect of different concentrations of trimethoprim (0, 0.003, 0.03, 0.2 mg L^-1^) and that of different pH levels. Block scores plots showing the distribution of samples with different concentrations at (a) pH 5 and (b) pH 7.

As there were multiple interactions, pH *versus* antibiotic level, MB-PCA was used to remove these potentially interacting factors. Fig 5 shows the results of MB-PCA and two block scores were derived for the two pH sub-groups. The distribution of samples exposed to different concentrations of trimethoprim at each pH are now clearly revealed in the 1^st^ PC and both pH 7 and 5 plots are congruent. The same process was repeated for the antibiotic dose effect (S6 Fig); the four block-scores were derived for drug dose based sub-groups, focusing upon this effect at two pH levels. The distribution of samples at pH 5 and 7 revealed a clear separation at 0.2 mg L^-1^, and partial separation at 0.03 and 0.003 mg L^-1^. Additionally, some separation can be seen in control samples, which is consistent with growth curves (S3 Fig), highlighting the varying phenotypic response to the different pH environments.

The loadings plots from all three chemometric analyses were complex (data not shown) and did not clearly reveal any obvious features. Indeed, the chemical resolution of IR spectroscopy is at the functional group level rather than at the level of specific metabolites and thus in order to study the subtle effects of trimethoprim on the intracellular metabolome of *E*. *coli* at pH 5, as well as more extreme effects at pH 7, a more sensitive and advanced analytical technique such as chromatography linked to mass spectrometry is required. It was expected that by including pH as well as sub-MIC antibiotic levels, we might be able to observe a wider response of *E*. *coli* to the drug in conditions similar to the pH range of urine, thus helping to elucidate the mechanism of action of the drug *in vivo*.

### Metabolic profiling of *E*. *coli* K-12 using GC-MS

**Fig 6:**
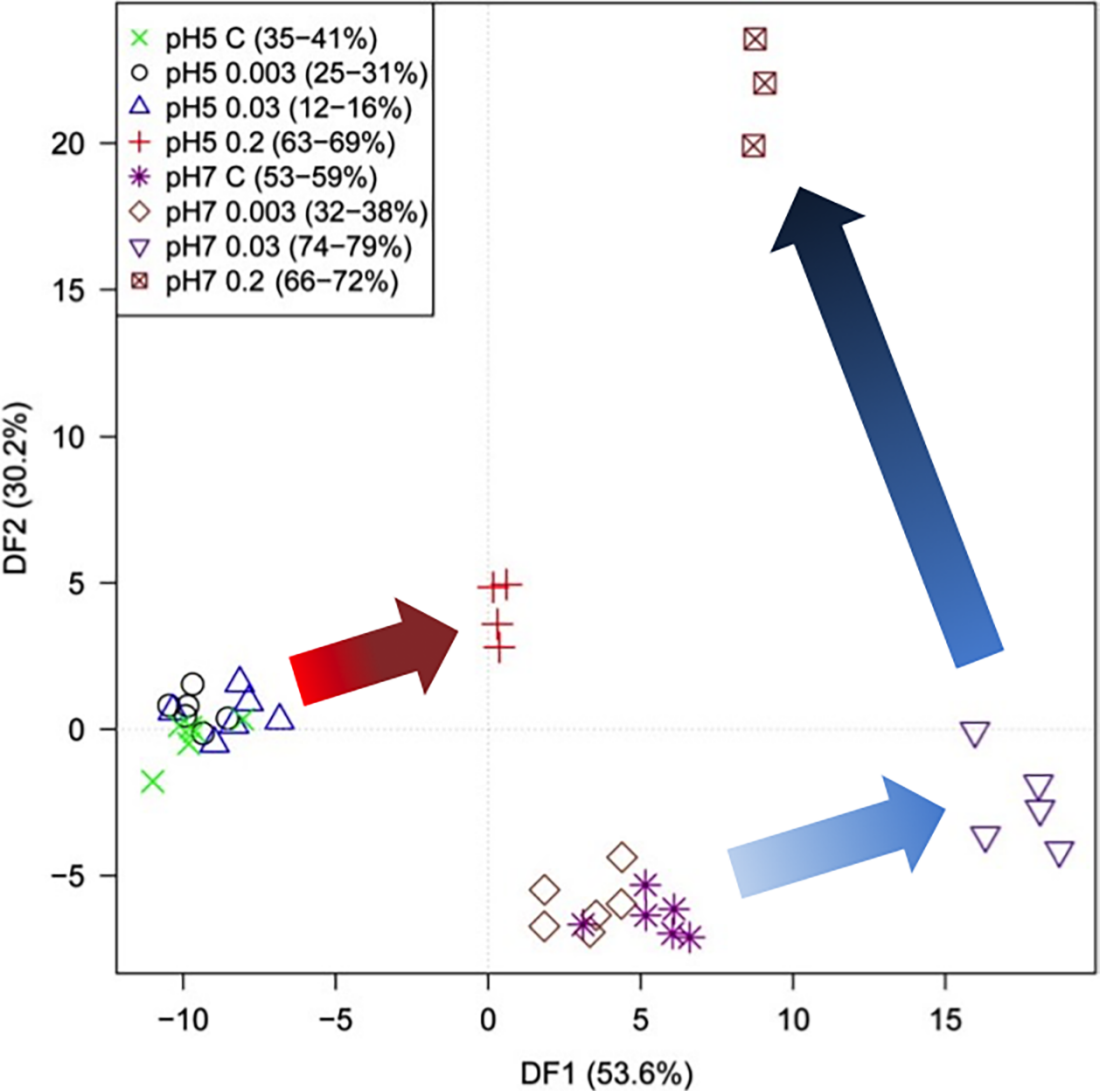
PC-DFA score plots of GC-MS profiles. 25 PCs were extracted from PCA and used as inputs to DFA, explaining 99% of the TEV. The legend in the figure shows the 95% CI for the correct classification of the 8 conditions. Significantly altered metabolites were mined through a combination of PC-DFA loadings and univariate significance testing (Student *t*-test). C, control.

The same bacterial samples analyzed by FT-IR spectroscopy were processed for GC-MS. For GC-MS all six biological replicates were analysed with a single technical replicate. Following MSI reporting standards for metabolite identification [33], 43 metabolites were identified at Level 1 (RI (+/─20 RI units) and MS matched to our in-house reference standard (80% similarity)), 20 were identified at Level 2 (putative MS match to external library (80% similarity)) and 4 at Level 3 (metabolite class indicated), while 92 were unknown (level 4) (see S1 Table for details of these metabolites and their relative abundance). GC-MS data were subjected to a multivariate analysis after data pre-processing. Initially, PCA was applied (data not shown) but unlike FT-IR spectroscopy, no separation was observed in the PCA scores plot and therefore PC-DFA was employed (Fig 6).

In this plot, clustering was apparent which was related to both pH effect and antibiotic dose effect, very similar to the class separation observed in the PC-DFA from the FT-IR data. Exposure to trimethoprim at pH 7 had more marked effect on intracellular metabolome compared to equivalent cells at pH 5 and antibiotic-related trajectories can be seen for both pH environments moving from 0 (control) through 0.003 and 0.03 to MIC levels at 0.2 mg L^-1^. The next stage was to relate the changes observed from GC-MS to the mechanisms of microbial response to pH and trimethoprim.

## Discussion

**Fig 7:**
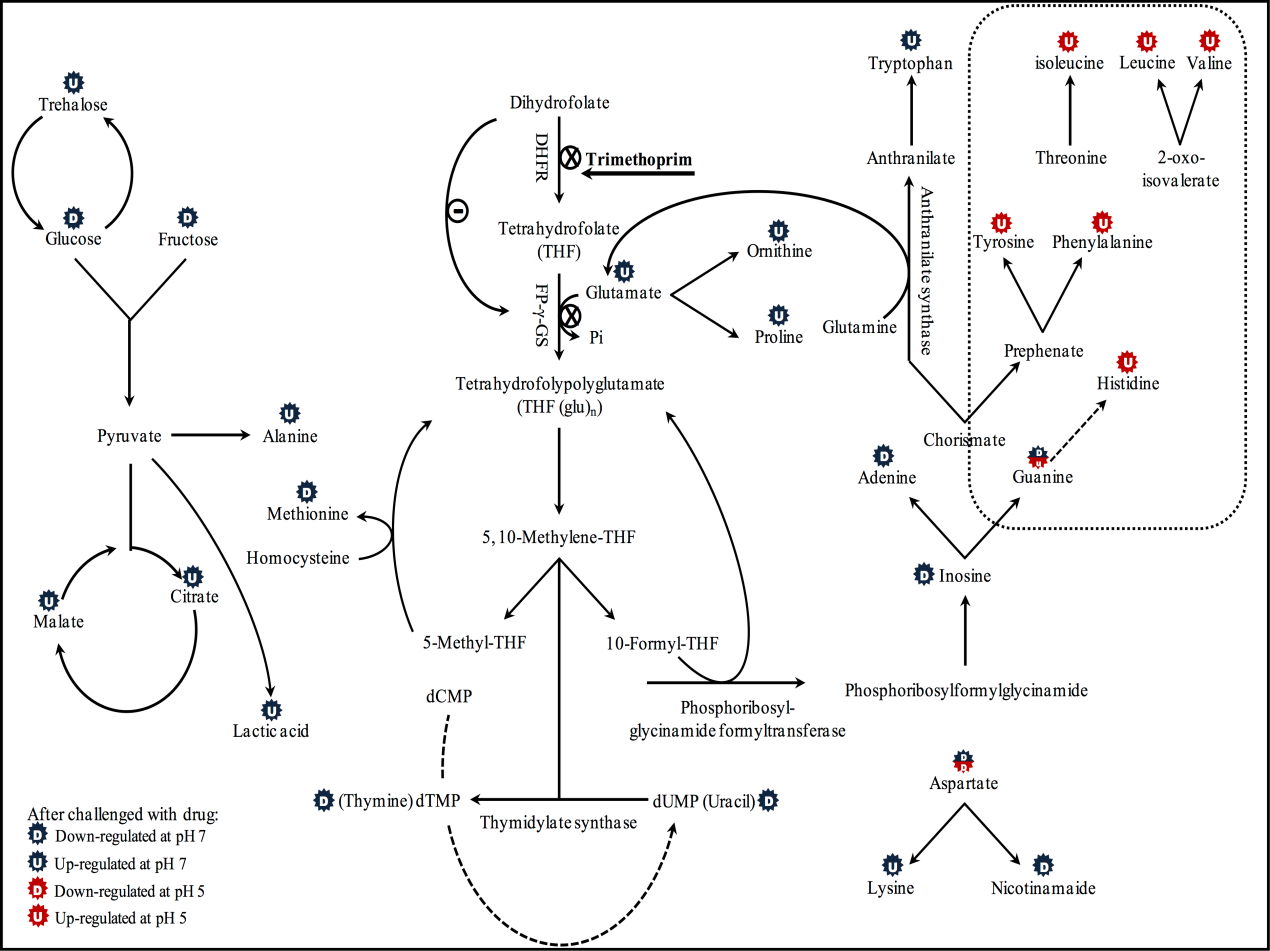
Metabolic effects of trimethoprim challenge on *E*. *coli* K-12 at pH 5 and pH 7. When partially ionized at pH 7, trimethoprim is seen to impact on metabolism directly associated with the dihydrofolate pathway, as well as off-target effects upon nucleotide, sugar and amino acid metabolism, glycolysis, the TCA cycle, and up-regulation of osmoprotectants. When trimethoprim is in a poorly ionized state (pH 5), it appears to have a profound effect upon the up-regulation of amino acid metabolism.

Different phenotypic responses to pH and trimethoprim exposure are expected to be observed. A summary of overall effects at both pH levels and changes in intracellular metabolites with respect to trimethoprim dose is shown in Fig 7. For full details of the relative metabolite levels, the reader is referred to S1 Table. S7 Fig provides an overlay of metabolite changes for all 8 conditions on the Kyoto Encyclopedia of Genes and Genomes (KEGG) pathway (Metabolic pathways- *Escherichia coli* K-12 MG1655) for the central metabolism of *E*. *coli* K-12 MG1655.

At pH 7, the permeability of the drug molecules is higher; metabolites linked with dihydrofolate reductase generally show a stronger response than metabolites extracted from samples incubated at pH 5 (see Fig 7). DHFR plays a key role in folate biosynthesis pathway. Therefore, a direct outcome of blocking DHFR is to deprive the cell of tetrahydrofolate (THF) and thus dihydrofolate accumulates. This in turn inhibits folylpoly-γ-glutamate synthetase (FP-γ-GS) [3]. This may indirectly result in the accumulation of glutamate, which would explain the rapid rise in the level of glutamate observed when the drug has its strongest activity (pH 7, 0.2 mg L^-1^) (S7 Fig). As detailed in EcoCyc, this non-essential amino acid is involved in numerous reactions including the biosynthesis of ornithine and proline. This explains the similarity in the levels of glutamate, ornithine and proline under all conditions which are at their highest levels under the same conditions; i.e. when the drug is very active (Fig 7). In ornithine biosynthesis, glutamate condenses with acetyl CoA to produce *N*-acetyl-glutamate, a precursor in ornithine synthesis [34]. Glutamate is involved in the biosynthesis of proline by being first phosphorylated to L-glutamate-5-phosphate and subsequent reduction to glutamate-5-semialdehyde, which is converted to pyrroline-5-carboxylate, which is then reduced to proline [35]. Proline acts as an osmoprotectant in bacteria [36], and it has been reported that glutamate also acts as an osmolyte in E. coli under specific growth conditions [37].

Trehalose, a disaccharide compound that consists of two glucose moieties, was first known as energy ‘storage’ metabolite, and later it was reported that trehalose also acts as protectant during adverse growth conditions in prokaryotic and eukaryotic cells [37]. Here, we find that under pH 7 and 0.2 mg L^-1^ trimethoprim conditions, the drug has its strongest effect on the cellular phenotype (S3 Fig) and this stress effect is reflected in the elevated trehalose levels observed (Fig 7 and S7 Fig). Under osmotic stress, it was reported that an osmotically regulated enzyme, trehalose phosphate synthase, is stimulated mainly by K^+^ and consumes glucose 6-phosphate and UDP-glucose to produce trehalose [38]. This could explain the concomitant reduction in the level of glucose and other sugars in general (Fig 7) when trehalose is elevated (S7 Fig). Alternatively, one or both of the trehalase anabolic enzymes (which are periplasmically and cytoplasmically located) that split trehalose into two glucose molecules are blocked or inhibited, and this would lead to an increase in the level of trehalose and reduce the pool of glucose available in the cell, thus obstructing glycolysis [39]. For sugars in general, the depletion of their levels after drug challenge may therefore be due to the stress of the drug, which increases catabolism and the consumption of sugars to generate a range of compatible solutes which act as osmoprotectants. A consequence of this reduction in sugars may in turn lead to an increase in the level of alanine, an amino acid that in higher organisms acts as a regulator in sugar metabolism and glycolysis [40] (Fig 7 and S7 Fig). In addition, this may simply be that carbon has been mobilized for (osmo)protection rather than being channelled directly into protein synthesis *per se*.

The direct effect of blocking dihydrofolate reductase, which is expected when the drug is near its MIC, is a reduction in THF. Consequently, there will be a depletion of THF-polyglutamate (THF (glu)_n_), a key metabolite in the biosynthesis of 10-formyl THF and 5,10-methylene THF, resulting in a reduction in these compounds (Fig 7); unfortunately, none of these metabolites were directly observed in our experiment as we conducted untargeted GC-MS rather than targeted LC-MS. The first of these compounds, 10-formyl THF, is a substrate of an enzyme called phosphoribosylglycinamide formyltransferase, which takes part in inosine monophosphate biosynthesis [41]. Reduction in this substrate results in a reduction in inosine monophosphate, which acts as a precursor of purine nucleotides, and thus results in a depletion of adenine and guanine [42] which we observe with untargeted GC-MS (Fig 7 and S7 Fig). Similarly, 5,10-methylene-THF is reduced to 5-methyl-THF which then methylates homocysteine to produce low levels of methionine, an essential amino acid that is converted to *N*-formyl-L-methionine, a starting amino acid in protein biosynthesis [42,43]. Methionine levels are also seen to decrease in our experiment (Fig 7). Methionine acts as a regulator of the first enzyme in its *de novo* biosynthesis (homoserine *O*-succinyltransferase), which produces *O*-succinyl-homoserine by transferring the succinyl group to homoserine from succinyl-CoA [43]. When methionine is at a low level (as found at high drug concentrations at pH 7), this may additionally result from extensive consumption in the feedback inhibition, possibly resulting in an accumulation of homoserine, which acts as a competitive inhibitor of glutamate dehydrogenase, an enzyme that has a role in a reversible reaction to produce and consume glutamate [44]. In addition, there is also a reduction of 5,10-methylene THF, which is catalyzed by thymidylate synthase to methylate deoxyuridine 5'-monophosphate (dUMP) and produces deoxythymidine 5'-monophosphate (dTMP). It has been reported that a reduction in dTMP results in a reduction of thymine [42,45], the latter is seen in our metabolic profiles (Fig 7 and S7 Fig). Unlike in higher organisms such as *Candidatus Phytoplasma mali*, the reaction mediated here by thymidylate synthase is currently thought to be irreversible and cannot therefore directly explain the reduction of dUMP [46]. Rather, a possible explanation is that in *E*. *coli* the deamination of deoxycytidine 5'-triphosphate (dCTP) to deoxyuridine 5'-triphosphate (dUTP) using deoxythymidine 5'-triphosphate (dTTP), which is reduced by the reduction in dTMP. This reduction results in the depletion of dUTP, which causes depletion in dUMP and thus in uracil [42] which we observe (Fig 7).

In general, all nucleotides were down-regulated with increasing antibiotic concentration, and this response was greater at pH 7 than at pH 5, and this is likely due to the high level of trimethoprim entering cells (Fig 3). Although guanine shows the same response, it has a unique response at pH 5 under high antibiotic dose, where its level increased considerably (Fig 7 and S7 Fig). We can find no explanation for this increase in guanine level under this condition. We also observe that many amino acids, including histidine, tyrosine, leucine, valine and phenylalanine (Fig 7, surrounded by a dotted rectangle), have the same response as guanine under this condition, including different levels and ratios compared with other conditions (S7 Fig). This may reflect a common feature among these metabolites which results in having almost the same response. For example, it was found that guanosine 5'-diphosphate 3'-diphosphate (ppGpp) is a histidine regulator in *Salmonella typhimurium*. This may explain why histidine and guanine gave similar responses under the eight conditions [47]. As for tyrosine, phenylalanine and tryptophan, it was found that these aromatic amino acids, which are the downstream products of a folate precursor called chorismate, gave the same response after 2 h of treatment [48]. However, in this experiment, when samples were collected after 18 h of drug exposure, only phenylalanine and tyrosine had the same response in that both accumulated most at the highest dose of the drug at pH 5, similar to histidine and guanine. By contrast, tryptophan accumulated at the same high dose at pH 7, where the drug is highly active and affects the growth of these bacteria. This shows that tyrosine and phenylalanine, the downstream products of prephenate, gave similar responses to guanine, unlike tryptophan (Fig 7), which had similar levels to alanine under all conditions (S7 Fig); this may be due to a common function or pathway between them [49]. Fig 7 (and S7 Fig) also shows that there is a correlation between tryptophan and glutamate, which is one of the products of tryptophan biosynthesis [50].

The branched chain amino acids valine, leucine and isoleucine have strongly interrelated biosynthetic pathways. Leucine and valine originate from 2-oxoisovalerate, while isoleucine originates from threonine (Fig 7) [42,51]. This explains their similar responses when challenged with trimethoprim at both pH levels (S7 Fig).

Turning to the detection of aspartic acid, its level decreased with increasing drug dose, regardless of pH. Phosphorylation of this amino acid is the starting point of synthesis of many amino acids including lysine, a basic amino acid that showed contrasting levels to those of its precursor aspartate at pH 7 [42]. Aspartate also acts as a precursor of nicotinamide, which showed strong depletion when the drug was highly active, perhaps because of an extensive use of its products, nicotinamide adenine dinucleotide (NAD) and nicotinamide adenine dinucleotide phosphate (NADP) which act as coenzymes [42]. Tryptophan was at high levels under the most extreme condition (0.2 mg L^-1^ trimethoprim, pH 7). Although there is evidence that tryptophan acts as a precursor in nicotinamide synthesis, the direct relationship between these two metabolites in *E*. *coli* is yet to be reported [42]. Nevertheless, quinolinate is one of the end products of tryptophan metabolism and is involved in nicotinamide metabolism, which may be taken as evidence of a correlation between these two metabolites in *E*. *coli* [52,53].

Alanine levels were observed to be high when the drug is highly active and the bacterium is under stress from exposure to trimethoprim. This may be correlated to extensive consumption of sugars, resulting in an increase in the level of pyruvate, an end-product of glycolysis. Pyruvate acts as a substrate of valine-pyruvate aminotransferase [54] and high levels of pyruvate result in an increase in alanine, an amino acid that acts as a regulator of sugar metabolism in higher organisms [40]. A potential consequence of the overflow of metabolism from the consumption of monosaccharides (Fig 7) is the excessive production of pyruvate generated via glycolysis. The cell would need to deal with this overproduction of pyruvate and this would in turn result in an increase in the level of lactic acid and tricarboxylic acid (TCA) cycle intermediates. We certainly observe a direct correlation of lactate to pyruvate (Fig 7 and S7 Fig) and the only two metabolites that we detected by GC-MS from the TCA cycle were citrate and malate, and both had their highest levels at pH 7 and 0.2 mg L^-1^ trimethoprim.

In conclusion, as well as measuring the direct effects on nucleotide metabolism that trimethoprim is known to cause we also observe pH dependent antibiotic effects on amino acid profiles and most significantly increased trehalose levels, an osmoprotectant that is produced when bacteria are under stress. These results provide a wider view of the action of trimethoprim, and metabolomics has also indicated several alternative areas of metabolism to be investigated further by time-course metabolic profiling, targeted metabolite quantification, and fluxomic-based investigation.

## Materials and methods

General maintenance and growth of *E*. *coli* K-12 MG1655 is provided in Supporting Information (S1 Text). This also includes preliminary investigations of growth optimization, different media and different pH levels. Details of the minimum inhibitory concentrations (MICs) calculation for trimethoprim at pH 5, 7 and 9 are also included.

### Antibiotic perturbation of *E*. *coli*

18 mL of LB medium at different pH: 5, 7 and 9, adjusted using NaOH or HCl, was inoculated with 1 mL of bacteria (Supporting Information) and 1 mL of 0.2, 0.03 and 0.003 mg L^-1^ of trimethoprim in 100 mL conical flasks. Control samples were identical except the 1 mL of trimethoprim was substituted with 1 mL of distilled water (dilution solvent). Each condition was replicated six times and each incubated for 18 h at 37°C and 200 rpm.

The overnight culture of each replicate was split for FT-IR and GC-MS to make sure that results were obtained from the same biological cultures. For FT-IR, 450 μL from each culture was collected and the biomass was washed three times with physiological saline and re-suspended in 400 μL of saline. For GC-MS, 15 mL was processed as described in Supporting Information.

In order to estimate the amount of trimethoprim inside the *E*. *coli* cells, cellular extracts were prepared, analyzed and quantified against a 20 point calibration curve constructed using a trimethoprim reference standard via LC-MS. For UHPLC-MS, a Thermo Accela UHPLC system (Thermo-Fisher Ltd.) coupled to a Thermo LTQ-Orbitrap XL MS system was employed (Thermo-Fisher). The methods used are described by Kim *et al*. [55]. Full details of methods are provided in S8 Fig for FT-IR and GC-MS and S9 Fig for LC-MS. In addition, the calibration curve for LC-MS is shown in Fig 2.

### FT-IR spectroscopy

Clean 96-well zinc selenide (ZnSe) plates (Bruker Ltd.) were used as sample carrier. 20 μL of the above bacterial preparations were spotted onto these plates and oven dried at 40°C for 45 min (as detailed by AlRabiah *et al*. [56]). High-throughput screening (HTS) FT-IR spectroscopic analysis was carried out on Bruker Equinox 55 infrared spectrometer (Bruker Ltd.) equipped with a HTX™ module according to the method of Winder *et al*. [57]. All spectra were obtained in the 4000–600 cm^-1^ range, 64 scans were acquired at 4 cm^-1^ resolution. These experimental conditions were maintained during all measurements.

After analysis, the FT-IR data were converted to ASCII format tab delimited files prior to data analysis in MATLAB 2010a (The Mathworks Inc.) and R version 2.13.1 (R Foundation for Statistical Computing). Prior to multivariate analysis (*vide infra*), CO_2_ signals were removed as detailed by AlRabiah *et al*. [56] and FT-IR data were baseline corrected using an extended multiplicative signal correction (EMSC) algorithm [58]. All data were subsequently autoscaled prior to analysis [59].

### GC-MS

For GC-MS, samples inoculated at pH 9 were excluded from the analysis due to the extreme effect of the drug at this pH that prevents the collection of adequate biomass for analysis. For the remaining conditions, 15 mL from each flask was collected and applied for further experiments (S8 Fig).

GC-TOF/MS was conducted using a LECO Pegasus III TOF/MS operated in GC-MS mode (Leco Corp.), with a Gerstel MPS-2 autosampler (Gerstel) and an Agilent 6890N GC × GC with a split/splitless injector and Agilent LPD split-mode inlet liner (Agilent Technologies). Full details of the GC-MS protocol used are provided in the Supporting Information and these follow the accepted Metabolomics Standards Initiative (MSI) guidelines [33] and follow our published protocols, and included pooled samples to act as quality controls (QC) [60,61]. The only difference in this study is that for metabolite extraction, 80% methanol was used rather than 100% methanol to enhance recovery of polar small molecules.

Following GC-MS, these data were processed using the deconvolution method reported by Begley *et al*. [61]. In addition, prior to statistical analysis, QC samples were used as in the work of Wedge *et al*. [62] to provide data quality assurance by evaluating and eliminating mass features that showed high deviation within QC samples.

### Metabolomics data analysis

For FT-IR and GC-MS, multivariate data analysis included unsupervised principal components analysis (PCA) and supervised principal components-discriminant function analysis (PC-DFA). PC-DFA depends on the prior knowledge of experimental structure (i.e. the experimental class structure) and a number of retained PCs to discriminate between groups (different classes). The PC-DFA models were validated via 1000 bootstrap cross-validations [63] and validation results are reported (as percentage of correct classification) inside the legends of the respective PC-DFA scores plot figures.

Additionally, for FT-IR spectroscopy, a multi-block (MB)-PCA model known as consensus PCA (CPCA) [64] was subsequently constructed to aid in spectral interpretation. The first CPCA model related the antibiotic dosing concentration trend for each pH condition as an individual block, and a second model was constructed to illustrate the distribution of samples at different pH levels between control samples and three different drug concentrations as individual blocks.

All multivariate data analyses were performed in R, and all scripts are available from the authors on request.

## Supporting information

S1 Text Supporting methods and results. Further description of methods and results.
(PDF)

S1 Table List of metabolites detected by GC-MS. Metabolites after extraction from control and stressed *E*. *coli* K-12 with trimethoprim at two different pH levels (5 and 7) are shown.
(XLSX)

S1 Fig Optical microscopic image of *E*. *coli* grown under different conditions. Magnification: ×100. E. coli K-12 inoculated in LB medium at different pH levels: (a) pH 5; (b) pH 7; (c) pH 9.
(PNG)

S2 Fig Growth curves of E. coli K-12 (at pH 7 in LB) exposed to different concentrations of trimethoprim. Blue indicates control samples (0 mg L-1); red 8 mg L-1; green 2 mg L-1; purple 0.3 mg L-1; turquoise 0.2 mg L-1; orange 0.03 mg L-1 and light blue 0.003 mg L-1.
(PNG)

S3 Fig Growth curves of E. coli K-12 exposed to different concentrations of trimethoprim. (a) Chemical structure of trimethoprim (blue circles show the main ionization points on the structure in acidic media). Blue indicates growth curves of control samples (0 mg L^-1^); red 0.003 mg L^-1^; green 0.03 mg L^-1^and purple 0.2 mg L^-1^ at (b) pH 9, (c) pH 7 and (d) at pH 5.
(PNG)

S4 Fig FT-IR spectra obtained from *E*. *coli* K-12. (a) After exposure to four concentrations of trimethoprim (0.2, 0.03, 0.003 and 0 mg L^-1^) at three different pH values (pH 5, 7 and 9). There were six biological replicates for each condition; each replicate was analysed three times, totalling 18 spectra for each condition (total number of spectra = 216). (b) After exposure to different concentrations of trimethoprim (0, 0.003, 0.03, 0.2 mg L^-1^) at pH 9 (total number of spectra = 72). (c) After exposure to different concentrations of trimethoprim (0, 0.003, 0.03, 0.2 mg L^-1^) at pH 7 (total number of spectra = 72). (d) After exposure to different concentrations of trimethoprim (0, 0.003, 0.03, 0.2 mg L^-1^) at pH 5 (total number of spectra = 72).
(PNG)

S5 Fig FT-IR spectra before and after CO_2_ removal and EMSC scaling. (a) FT-IR spectra obtained from *E*. *coli* K-12 after exposure to four concentrations of trimethoprim (0.2, 0.03, 0.003 and 0 mg L^-1^) at two different pH values (5 and 7). (b) FT-IR spectra post CO_2_ removed at ≈ 2350 cm^-1^ and EMSC scaling.
(PNG)

S6 Fig FT-IR multi-block PCA scores plot. The plot shows the distribution of samples with different pH levels at different drug concentrations: (a) 0 mg L^-1^, (b) 0.003 mg L^-1^, (c) 0.03 mg L^-1^ and (d) 0.2 mg L^-1^.
(PNG)

S7 Fig KEGG metabolic pathway of *E*. *coli* K-12 MG1655. The map highlights significant metabolites with their relative levels subjected to different concentrations of trimethoprim at different pH levels.
(PNG)

S8 Fig General scheme of sample preparation for FT-IR and GC-MS. Sample preparation includes: (1) analysis by Bioscreen to determine the MIC of trimethoprim and produce the growth curves of *E*. *coli* K-12 at pH 5 and 7 with and without drug challenge. (2) FT-IR analysis of samples after washing with normal saline. (3) GC-MS analysis of samples after quenching and extraction using 60% and 80% cold (-48°C) methanol respectively.
(PNG)

S9 Fig General scheme of sample preparation for LC-MS. Sample preparation includes: (1) analysis by Bioscreen to produce the growth curves of E. coli K-12 at pH 5 and 7 after challenge with 0.8 mg L-1 of trimethoprim added at two time points: (I) at the beginning of the lag phase (time = 0 h) and (II) at the mid-exponential phase (time = 5 h). (2) LC-MS analysis of sample extracts, for relative quantification of the intracellular drug levels after quenching and extraction using 60% and 80% cold (-48°C) methanol respectively.
(PNG)
