# Supplementary material for: pH plays a role in the mode of action of trimethoprim on Escherichia coli
Source: PLoS One. 2018 Jul 13;13(7):e0200272. doi: 10.1371/journal.pone.0200272 (PMC6044521; doi:10.1371/journal.pone.0200272)
Supplement: S1 Text — Further description of methods and results. (PDF) [file pone.0200272.s001.pdf]

Supporting Methods

General chemicals

Unless otherwise stated, all chemicals were attained from Fisher Scientific Ltd., and all solvents and acids were obtained from Sigma-Aldrich.

Microorganisms

Escherichia coli K-12 (MG1655) was used for this investigation and its genome is available [1].

Media

Nutrient agar (NA) was prepared from a preparatory mixture (beef extract 3 g L^{-1}, peptone 5 g L^{-1}, NaCl 8 g L^{-1} and 12 gL^{-1} of agar no. 2) (Lab-M) following the manufacturer's instructions (28 g in 1 L of deionized water) and then autoclaved (at 121 ºC and 15 psi for 15 min).

Lysogeny broth (LB) was prepared by dissolving 10 g of tryptone (Formedia), 5 g of yeast extract (Amersham Life Sciences) and 10 g of NaCl in 1 L of reverse osmosis water and after this was autoclaved (121 ºC, 45 min and 15 psi).

Phage broth (ψ) was prepared by dissolving 0.5 g $MgSO_4.7H_2O$ (VWR International Ltd, Lutterworth, UK), 0.74 g $CaCl_2.2H_2O$ (VWR International Ltd.), 1 g glucose, 5 g tryptone (Oxoid Ltd.), 5 g yeast extract (Oxoid Ltd.) and 5 g Lab Lemco Powder (Oxoid Ltd.) in 800 mL of distilled water then adjusting to pH 7.2 with NaOH. This was made up to 1 L with distilled water and finally autoclaved (121 ºC, 45 min, 15 psi).

Nutrient broth (NB) was prepared from a preparatory mixture (beef extract 1 g L^{-1}, yeast extract 2 g L^{-1}, peptone 5 g L^{-1}, NaCl 5 g L^{-1}) (Lab-M) following the manufacturer's instructions (13 g in 1 L of H_2O) and then autoclaved (121 ºC, 45 min, 15 psi).

In order to calculate the minimum inhibitory concentration (MIC) of trimethoprim (Sigma-Aldrich), 100 mg was dissolved in 50 mL distilled water and 500 µL glacial acetic acid [2]. A series of trimethoprim dilutions (10, 8, 4, 2, 1.5, 1.4, 1.3, 1.2, 1.1,

1, 0.9, 0.8, 0.7, 0.6, 0.5, 0.4, 0.3, 0.2, 0.1, 0.09, 0.08, 0.06, 0.05,0.03, 0.02, 0.003, 0.002 mg L^{-1}) were then prepared with distilled water [2]. MIC is defined as "the lowest concentration of the agent that completely inhibits the growth of the test organism" [3]. Alternatively, it can be defined as the concentration of an agent producing 90% inhibition of the original inoculum [4]. Within this chapter, the first definition of MIC applies since the experimentally determined MIC was based upon 100% inhibition.

Growth characteristics

E. coli was streaked on a NA plate to obtain axenic colonies. Biomass was collected from single colonies to prepare 1 mL 20% (v/v) glycerol working inoculum stocks which were stored at –20 ºC. A 1 mL master stock of bacteria was also stored at –80 ºC (S8 Fig.).

Gram staining was performed using a Sigma-Aldrich kit, the stained bacterial cells were observed with a Zeiss LSM 510 META confocal microscope using the ×100 objective (Carl Zeiss Ltd.).

Starting growth condition. At the start of each experiment, 49 mL of medium was inoculated with 1 mL of working stock and incubated at 37 ºC in a shaking incubator at 200 rpm for 24 h. The overnight cultures (1 mL) were diluted with 49 mL fresh media and further incubated at 37 ºC, 200 rpm for 1 h. These new cultures were diluted with physiological saline (0.9% (w/v) NaCl) to 0.5 McFarland standard optical density (OD) at 600 nm using a Biomate 5 spectrophotometer (Thermo) and used as experimental inoculate (S8 Fig.).

Estimation of bacterial biomass. In order to standardise the size of the inocula for growth curve experiments, 40 mL of the culture was diluted and washed two times with physiological saline (0.9% (w/v) NaCl). The bacterial turbidity was adjusted to 0.5 McFarland standard (OD 0.1 ± 0.02) optical density (OD) at 600 nm using a Biomate 5 (S8 Fig.). 0.5 McFarland standard was prepared by adding 85 mL of 1% (w/v) H_2SO_4 to 0.5 mL of 1.175% (w/v) barium chloride dihydrate ($BaCl_2.2H_2O$), and made up to 100 mL with deionized water and mixed well.

The bacterial growth curves were measured using an OD 600 nm in a Bioscreen spectrophotometer (Labsystems). This Bioscreen was run at the following settings: 10 min preheating, incubation temperature 37 ºC, continuous medium shake, measurement interval 10 min and 24 h for the total experiment.

180 µL of LB at pH 5 and 7 (± 0.2) was inoculated with 10 µL of 0.5 McFarland standard inocula and 10 µL serial dilutions of trimethoprim in Bioscreen plates. The control samples consisted of 180 µL of LB at pH 5 and 7 (± 0.2) with 10 µL of 0.5 McFarland standard inocula and 10 µL distilled water. Incubation was for 18–24 h, as described above. Four pilot experiments were applied on *E. coli* K-12.

Each class in all Bioscreen experiments was prepared in five biological replicates, and all growth was conducted at 37 °C for 24 h.

Pilot experiments

1. Comparison of growth curves between the different media. 190 µL of LB, ψ and NB media were inoculated with 10 µL of 0.5 McFarland standard inocula Bioscreen plates.

2. Comparison of growth curves in LB media at pH 3, 5, 7 and 9. 190 µL of LB at different pH 3, 5, 7 and 9 (± 0.2) were inoculated with 10 µL of 0.5 McFarland standard inocula in Bioscreen plates.

3. Determination of the drug MIC in neutral LB media (pH 7). 180 µL of LB were inoculated with 10 µL of 0.5 McFarland standard inocula and 10 µL serial dilutions of trimethoprim in Bioscreen plates. The control samples consisted of 180 µL of LB with 10 µL of 0.5 McFarland standard inocula and 10 µL of distilled water.

4. Challenge of E. coli with trimethoprim at different pH levels. 180 µL of LB at pH 5, 7 and 9 (± 0.2) were inoculated with 10 µL of 0.5 McFarland standard inocula and 10 µL serial dilutions of trimethoprim in Bioscreen plates. The control samples consisted of 180 µL of LB at pH 5, 7 and 9 (± 0.2) with 10 µL of 0.5 McFarland standard inocula and 10 µL of distilled water.

GC-MS

Chemicals for GC-MS analysis. All materials were purchased from Sigma-Aldrich unless otherwise stated. Pyridine (extra dry), hexane, methoxylamine hydrochloride, and *N*-methyl-*N*-trimethylsilyl-trifluoroacetamide (MSTFA) were obtained from Acros Organics (Loughborough, UK). The internal standards malonic acid-d_2, succinic acid-d_4, glycine-d_5 and leucine-d_4 were purchased from Sigma-Aldrich, as were methanol and water, which were of analytical grade or higher purity.

Sample preparation. Samples for FT-IR spectroscopy and GC-MS were collected from the same respective cultures for each sample. For GC-MS, samples inoculated at pH 9 were excluded from the analysis due to the extremely strong effect of the drug at this pH level that prevents the collection of adequate biomass for analysis. For the remaining conditions, 15 mL from each flask was collected and applied for further experiments (S8 Fig.).

Metabolic Quenching. 15 mL from each flask of the overnight culture was collected and added to a double volume of 60% cold methanol (–48 ºC) and mixed quickly [5]. The quenched culture was centrifuged for 10 min at 4800 *g* and –8 ºC. 2 mL of the supernatant was collected to assess the leakage of internal metabolites and the remainder was removed rapidly. Further centrifugation was applied on the pellet for 2 min to remove further residual supernatant. The cell pellets and collected supernatants were stored at –80 ºC until metabolite extraction and further analysis (see S8 Fig.).

Metabolite extraction. Methanol was applied as the extraction solvent. The biomass pellets were suspended in 1 mL of 80% methanol (–48 ºC), transferred to 2 mL tubes, flash frozen in liquid nitrogen and placed on wet ice, once semi-defrosted the samples were vortexed thoroughly for approximately 30 s.

The freeze-thaw and vortex cycle was repeated further two times to maximise extraction of intracellular metabolites from within the cells. The suspensions were centrifuged for 5 min at 13000 *g* and –9 ºC. The supernatants were retrieved to clean 2 mL tubes and kept on dry ice. 500 µL of 80% methanol (–48 ºC) was added to the pellet and the whole procedure was repeated and the second extraction aliquot was

combined with the first (on dry ice) and vortexed thoroughly (S8 Fig.). For GC-MS samples, 825 µL of each extract (normalised to OD and made up with 80% methanol) was spiked with 100 µL of internal slandered (0.3 mg L^{-1} succinic-d_4 acid, malonic-d_2 acid and glycine-d_5 in HPLC grade water). Quality control (QC) samples were created by combining 60 µL from each sample and mixing thoroughly, the QC mix was divided into 4 QC samples, each containing 606 µL of the QC mix (the OD averaged volume for all experimental replicates). All samples were dried for 16 h using a speed vacuum concentrator operated at 30 ºC (Eppendorf 5301 concentrator).

GC-MS sample derivatisation. A two-step chemical derivatisation was used, due to the non-volatile nature of many metabolite classes within central metabolism. Before analysis of the reaction products with GC-MS, firstly carbonyl moieties were substituted via methoxyamination and secondly there was a silylation reaction. In order to remove residual condensation, samples removed from −80 ºC storage were placed in a speed vacuum concentrator for 30 min. Following this, the extracts were dissolved in 50 µL of 20 mg mL^{-1} *O*-methoxylamine hydrochloride in pyridine. Then, they were vortexed and incubated at 60 ºC for 30 min in a dry-block heater. The next stage was to add 50 µL of MSTFA and the extracts were mixed again and incubated at 60 ºC for 30 min. Once that stage was complete, 20 µL of retention index marker solution was added (0.3 mg mL^{-1} docosane, nonadecane, decane, dodecane, and pentadecane in pyridine) before 15 min of centrifugation at 15800 *g*. The supernatant (100 µL) that was obtained from this process was then transferred to 2 mL amber glass GC-MS vials fitted with 200 µL inserts prior to analysis. With the employed GC-MS analytical method, a throughput of 40 samples per day is possible, so to achieve a higher level of sample chemical stability, randomised batches of 40 samples per day were derivatised across the analysis period with QC samples also derivatised across multiple batches so as to provide a measure of derivatisation (technical) and instrument (analytical) error.

Instrumentation. In order to achieve unbiased statistical analysis, it was important to prevent analytical drift in relation to the sample experimental class. This was done by analysing the samples over separate randomised analytical blocks. The QC samples were analysed across the analytical batches at regular intervals. To perform

initial column conditioning to the sample matrix, five injections of QC sample were performed, followed by five injections of experimental samples and then a QC injection. At the end of the block run, once all experimental samples had been analysed, three QC injections were made. This procedure was repeated for each daily block of samples (‘derivatisation blocks’) until sample analysis was completed. GC-TOF/MS system stability was assessed across both derivatisation batches ran as a single experimental block. This was facilitated by calculating the relative standard deviations (RSDs) of each feature detected within the QC samples and removal of metabolite features showing greater than a 25% deviation, deemed as unreliably detected features. In accordance with the methods of Begley *et al.* [6] and Wedge *et al.* [7], the equipment used for the GC-TOF/MS analyses included a LECO Pegasus III TOF/MS operated in GC-MS mode (Leco Corp.), with a Gerstel MPS-2 autosampler (Gerstel) and an Agilent 6890N GC × GC with a split/splitless injector and Agilent LPD split-mode inlet liner (Agilent Technologies). A 30 m × 0.25 mm × 0.25 µm VF17-MS bonded phase capillary column (Varian) was used at a constant helium carrier gas flow of 1 mL min^{-1}. The temperature program was as follows: 4 min hold at 70 ºC, 20 ºC min^{-1} to 300 ºC, 4 min hold. A split ratio of 4:1 was used for sample injections of 1 µL. The operational temperature of the injector was 280 ºC, and after 30 s, a 25 mL min^{-1} gas saver flow was used, and the transfer line was held at 240 ºC. The mass spectrometer had a source temperature of 220 ºC and was operated at 70 eV ionization energy, and acquired *m*/*z* 45–600 at 20 Hz.

GC-TOF/MS data pre-processing and analysis. Raw GC-TOF/MS data was processed using the exact method of Begley *et al.* [6] and Wedge *et al.* [7], which was based on the ‘Compare’ capability of LECO’s ChromaTOF v3.25 software (Leco Corp.). In this method, a set of reference spectra are compiled for a list of QC representative metabolites, including QC samples from within each analytical block. All later samples are then searched against the particular reference table that has been created. For the reference table to be unbiased, all peaks evident in a representative QC sample that were within an appropriate range for signal/noise (S/N) ratio and chromatographic peak width could be included. Peak identities were assigned, whenever possible, based on similarity matching to mass spectral entries in the NIST (National Institute of Standards and Technology) library and GMD (Golm

Metabolome Database) [8] for putative level identification or by comparing the mass spectral and retention index with an in-house generated metabolite library of authentic reference compounds. A peak width of 1.8 s and a minimum S/N ratio of 10 were applied as the peak detection parameters in ChromaTOF. Relative quantification for each metabolite was applied on the basis of internal standards, and retention indices were calculated using retention markers. The quantified peak areas for each metabolite within each sample, along with their metabolite identities and the sample information, were transferred to an XY matrix in Microsoft Excel. Prior to statistical analysis, QC samples were used as in the work of Wedge *et al.* [7] to provide data quality assurance by evaluating and eliminating mass features that showed high deviation within QC samples.

Trimethoprim quantification from *E. coli* using LC-MS

All materials were purchased from Sigma-Aldrich unless otherwise stated.

Sample preparation. 18 mL of LB medium at pH 5 and 7 in 100 mL conical flasks was inoculated with 1 mL of 0.5 McFarland standard inoculum and 1 mL of 0.8 mg L^{-1} of trimethoprim (four times higher than the MIC) added at two time points (time = 0 and 5 h). Control samples were identical except the 1 mL of trimethoprim was substituted with 1 mL of distilled water (dilution solvent of the drug) at the beginning of the experiment. In this experiment, each condition was replicated three times except the control samples which were prepared in six replicates to use three replicates to spike with drug after extraction (explained further below). All samples were incubated for 6 h at 37 ºC and 200 rpm. 15 mL from each flask were collected and were applied for further experiments (S9 Fig.).

Trimethoprim extraction. Quenching metabolism was identical to that used for the GC-MS samples (see above).

The metabolite and drug extraction was similar to that applied for GC-MS samples. However, for LC-MS samples, instead of normalisation of the samples to the OD 600 reading by dilution with 80% methanol prior to sample drying, the samples were prepared and analysed in an identical fashion, and the detected relative concentration levels of trimethoprim were then normalised to the OD 600 reading.

Instrumentation. For LC-MS analysis, the samples were reconstituted in 100 μL of water, vortex mixed and centrifuged for 15 min at 10000 *g*. The supernatants were transferred to analytical vials with 200 μL fixed inserts, stored in the autosampler at 5 ºC and analysed within 24 h of reconstitution in LC-MS positive ionization mode. Ultra high performance LC separations were performed according to the following method on a Thermo Accela UHPLC system. A Hypersil Gold C_{18} reversed phase column (100 mm × 2.1 mm × 1.9 μm) was used (Thermo-Fisher Ltd.). The UHPLC was operated at a flow rate of 400 μL min^{-1}, the column was maintained at a temperature of 50 ºC. Solvent A (HPLC water and 0.1% formic acid) and solvent B (HPLC methanol and 0.1% formic acid) gradient programme was as follows: 100% A 0–1 min, 100% A–100% B 1–12 min, 100% B 12–20 min, 100% A 20–22 min. Prior to sample analysis, a new LC column was conditioned with the linear gradient conditions for 40 min at a flow rate of 400 μL min^{-1}. A sample injection volume of 10 μL was employed in wasteless mode. After each sample analysis, the UHPLC system was conditioned with the initial gradient solvent conditions thus returning the system to a clean state prior to the analysis of the next sample. Autosampler syringe and line washes were performed with 80% methanol. The Thermo LTQ-Orbitrap XL MS system was operated using Xcalibur software (Thermo-Fisher Ltd.) exactly following the method described by Dunn *et al.* [9]. Prior to the analytical batch runs, the LTQ and Orbitrap were tuned to optimize conditions for the detection of ions in the mid detection range of *m/z* 100−1000 and calibrated according to the manufacturer's predefined methods in both ESI polarities with the manufacturer's recommended calibration mixture, consisting of caffeine, sodium dodecyl sulfate, sodium taurocholate, the tetrapeptide MRFA and Ultramark 1621. The Orbitrap was operated in full scan mode at a mass resolution of 30000 (FWHM defined at *m/z* 400) and a scan speed of 0.4 s. The ESI conditions were optimized to allow efficient ionization and ion transmission of trimethoprim without causing insource fragmentation leading to the detection of an intact parent mass ion ($M+H^+$) with high sensitivity.

A trimethoprim reference standard was run for the purpose of constructing a twenty point calibration curve (0.05, 0.06, 0.08, 0.09, 0.1, 0.2, 0.3, 0.4, 0.5, 0.6, 0.7, 0.8, 0.9, 1, 1.1, 1.2, 1.3, 1.4, 1.5, 1.8 mg L^{-1}) (Fig. 2). The response of the reference standard was compared between the reference standard in solution and also when spiked into

an intracellular sample matrix, no major changes in the measured peak area for trimethoprim ($M+H^+$ *m/z* 291.143) were observed. The intracellular samples and reference standards were run in a completely randomised order. Blank samples were interspersed throughout the analytical run to allow assessments of carry over to be made. The peak areas were calculated for the extracted ion chromatogram of trimethoprim ($M+H^+$ *m/z* 291.143) and imported into Microsoft Excel.

Data processing. Using Microsoft Excel 2007, the peak areas obtained for the trimethoprim reference standard were plotted against the known concentration level, thus producing a calibration curve. The extracted peak areas for trimethoprim within the intracellular extracts were then inferred against the calibration curve, thus revealing the concentration of trimethoprim within the intracellular extract. As a final step, the intracellular concentration levels were then normalised to the OD 600 reading taken from the original bacterial culture, thus providing a normalised non-biased relative quantification value to compare between the intracellular extracts and experimental conditions.

References

1. Blattner FR, Plunkett G, Bloch CA, Perna NT, Burland V, Riley M et al. The complete genome sequence of *Escherichia coli* K-12. Science. 1997;277, 1453-1462.
2. Andrews JM. Determination of minimum inhibitory concentrations. J Antimicrob Chemoth. 2001;48: 5-16.
3. Madigan M, Marktinko J, Parker J. Brock biology of microorganisms. London: Prentice Hall Internationall; 2000.
4. Aagaard J, Gasser T, Rhodes P, Madsen PO. MICs of ciprofloxacin and trimethoprim for *Escherichia coli*: influence of pH, inoculum size and various body fluids. Infection. 1991;19, S167-S169.
5. Winder CL, Dunn WB, Schuler S, Broadhurst D, Jarvis R, Stephens GM et al. Global metabolic profiling of *Escherichia coli* cultures: an evaluation of methods for quenching and extraction of intracellular metabolites. Anal Chem. 2008;80: 2939-2948.
6. Begley P, Francis-McIntyre S, Dunn WB, Broadhurst DI, Halsall A, Tseng A et al. Development and performance of a gas chromatography−time-of-flight mass spectrometry analysis for large-scale nontargeted metabolomic studies of human serum. Anal Chem. 2009;81: 7038-7046.
7. Wedge DC, Allwood JW, Dunn W, Vaughan AA, Simpson K, Brown M et al. Is serum or plasma more appropriate for intersubject comparisons in metabolomic studies? An assessment in patients with small-cell lung cancer. Anal Chem. 2011;83: 6689-6697.
8. Kopka J, Schauer N, Krueger S, Birkemeyer C, Usadel B, Bergmuller E et al. GMD@CSB.DB: the Golm metabolome database. Bioinformatics. 2005;21, 1635-1638.
9. Dunn WB, Broadhurst D, Brown M, Baker PN, Redman CWG, Kenny LC et al. Metabolic profiling of serum using ultra performance liquid chromatography and the LTQ-Orbitrap mass spectrometry system. J Chromatogr B. 2008;871: 288-298.
