# Supplementary figures and images for: pH plays a role in the mode of action of trimethoprim on Escherichia coli
Source: PLoS One. 2018 Jul 13;13(7):e0200272. doi: 10.1371/journal.pone.0200272 (PMC6044521; doi:10.1371/journal.pone.0200272)

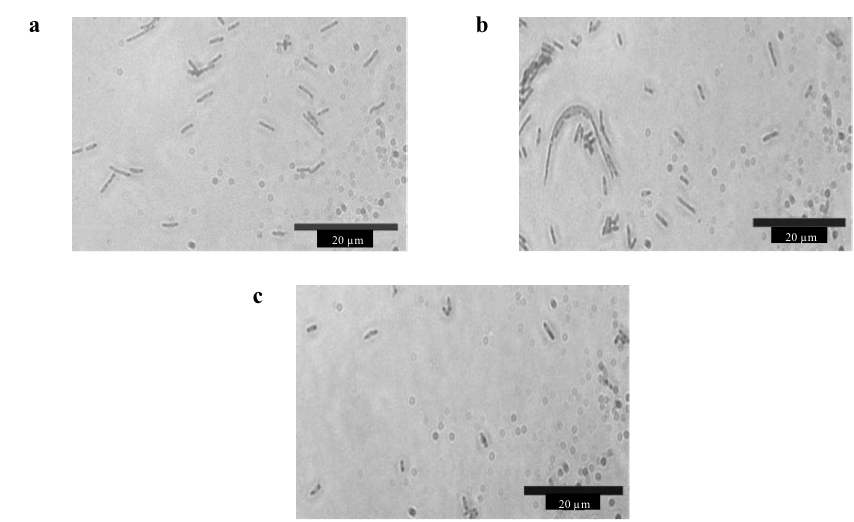

Supplement: S1 Fig — Magnification: ×100. E. coli K-12 inoculated in LB medium at different pH levels: (a) pH 5; (b) pH 7; (c) pH 9. (PNG) [file pone.0200272.s003.png]

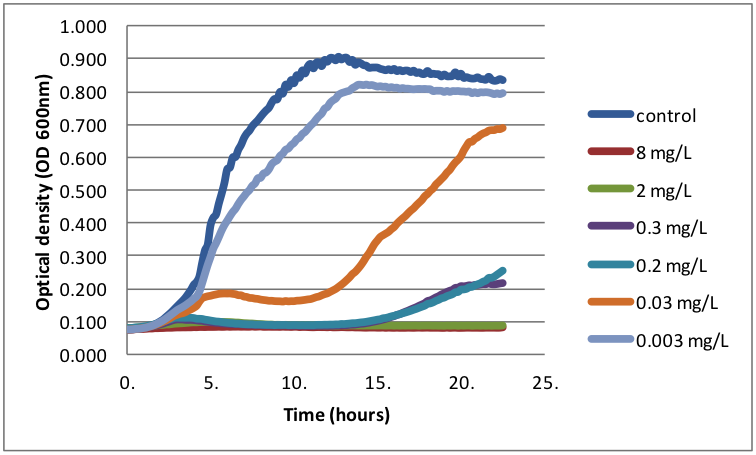

Supplement: S2 Fig — Blue indicates control samples (0 mg L-1); red 8 mg L-1; green 2 mg L-1; purple 0.3 mg L-1; turquoise 0.2 mg L-1; orange 0.03 mg L-1 and light blue 0.003 mg L-1. (PNG) [file pone.0200272.s004.png]

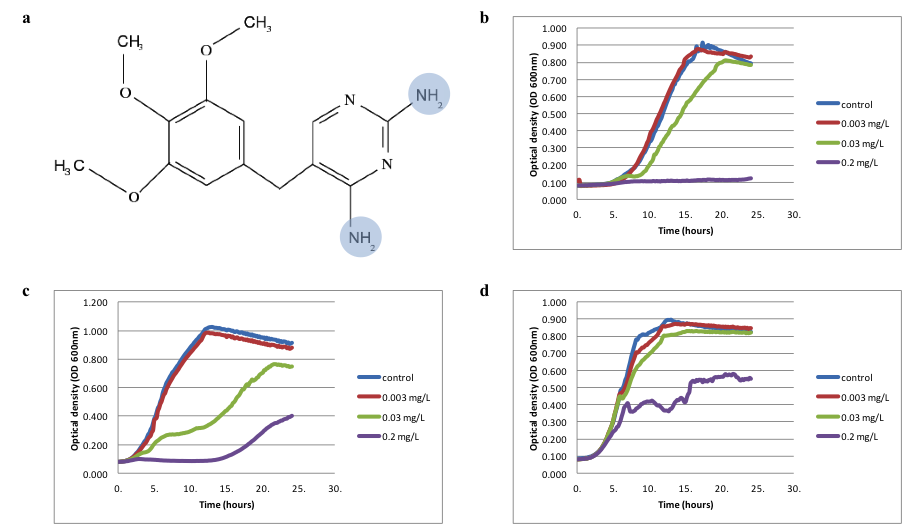

Supplement: S3 Fig — (a) Chemical structure of trimethoprim (blue circles show the main ionization points on the structure in acidic media). Blue indicates growth curves of control samples (0 mg L-1); red 0.003 mg L-1; green 0.03 mg L-1and purple 0.2 mg L-1 at (b) pH 9, (c) pH 7 and (d) at pH 5. (PNG) [file pone.0200272.s005.png]

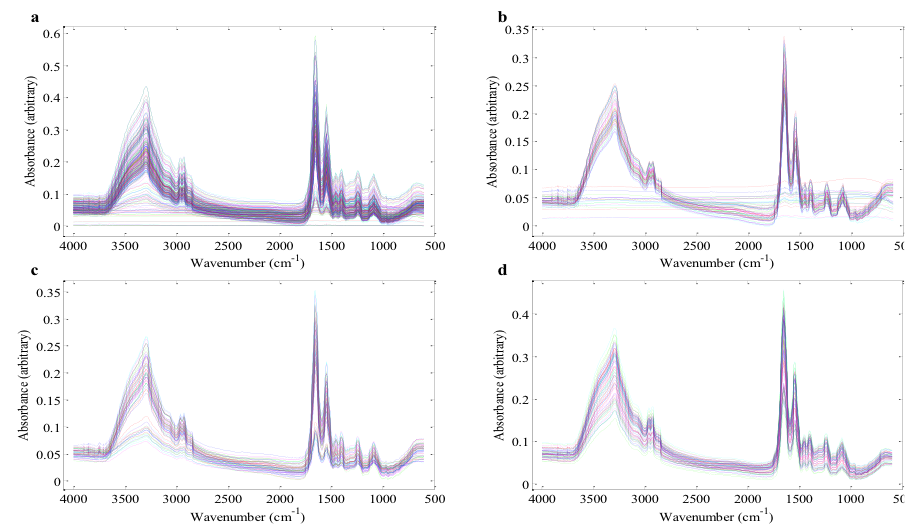

Supplement: S4 Fig — (a) After exposure to four concentrations of trimethoprim (0.2, 0.03, 0.003 and 0 mg L-1) at three different pH values (pH 5, 7 and 9). There were six biological replicates for each condition; each replicate was analysed three times, totalling 18 spectra for each condition (total number of spectra = 216). (b) After exposure to different concentrations of trimethoprim (0, 0.003, 0.03, 0.2 mg L-1) at pH 9 (total number of spectra = 72). (c) After exposure to different concentrations of trimethoprim (0, 0.003, 0.03, 0.2 mg L-1) at pH 7 (total number of spectra = 72). (d) After exposure to different concentrations of trimethoprim (0, 0.003, 0.03, 0.2 mg L-1) at pH 5 (total number of spectra = 72). (PNG) [file pone.0200272.s006.png]

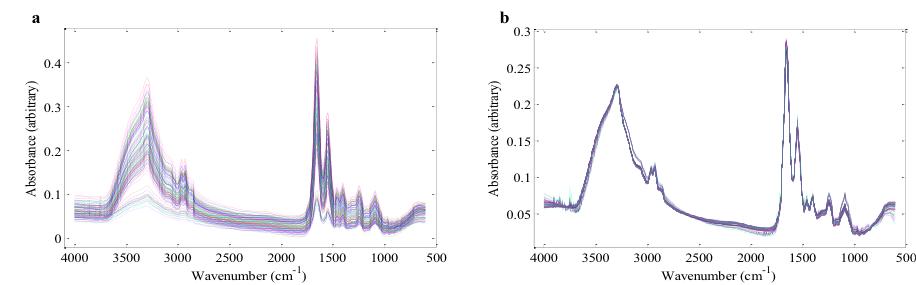

Supplement: S5 Fig — (a) FT-IR spectra obtained from E. coli K-12 after exposure to four concentrations of trimethoprim (0.2, 0.03, 0.003 and 0 mg L-1) at two different pH values (5 and 7). (b) FT-IR spectra post CO2 removed at ≈ 2350 cm-1 and EMSC scaling. (PNG) [file pone.0200272.s007.png]

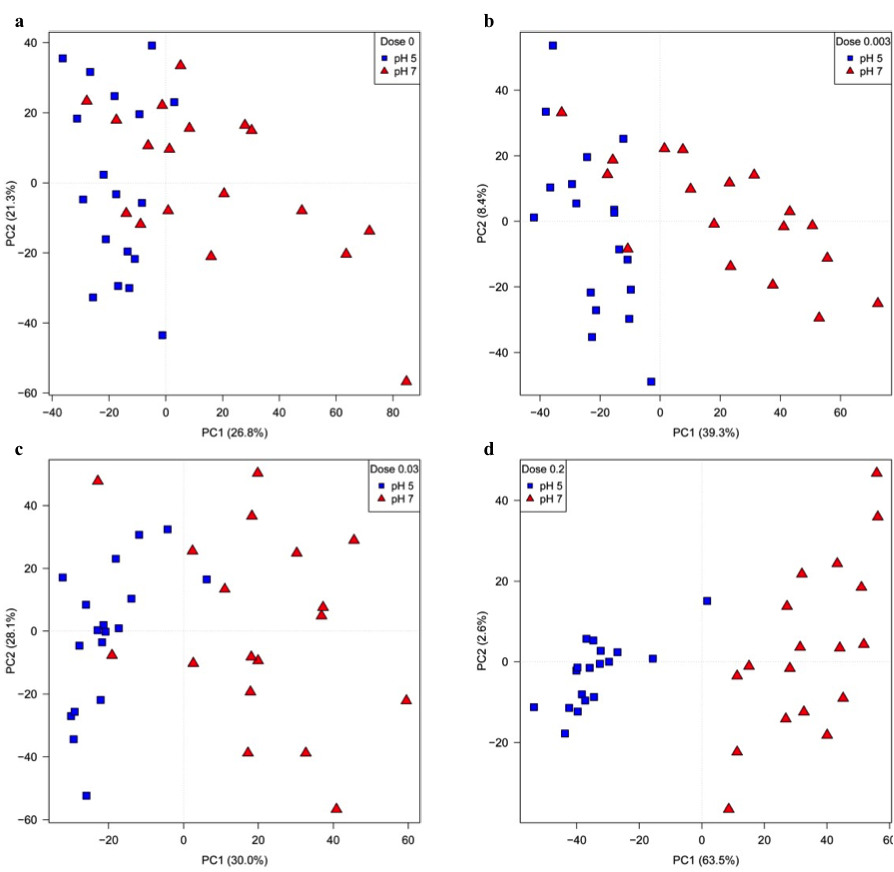

Supplement: S6 Fig — The plot shows the distribution of samples with different pH levels at different drug concentrations: (a) 0 mg L-1, (b) 0.003 mg L-1, (c) 0.03 mg L-1 and (d) 0.2 mg L-1. (PNG) [file pone.0200272.s008.png]

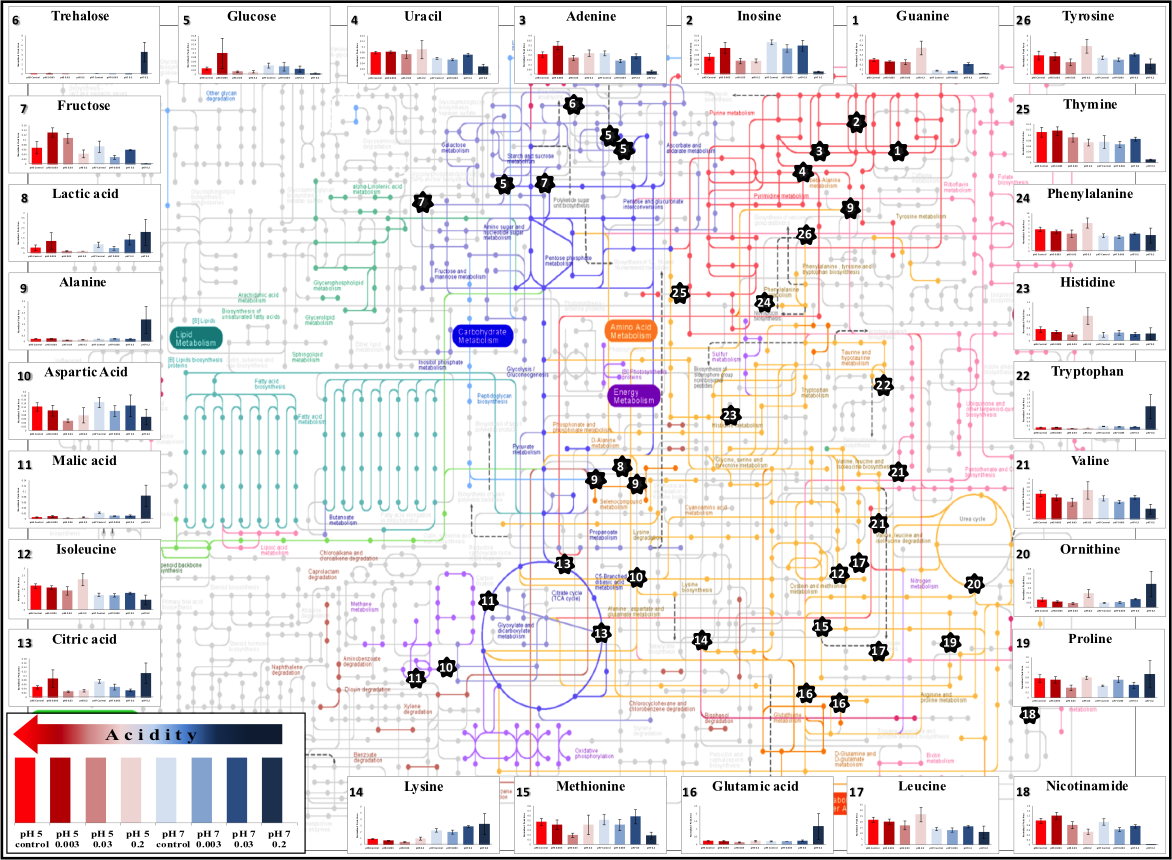

Supplement: S7 Fig — The map highlights significant metabolites with their relative levels subjected to different concentrations of trimethoprim at different pH levels. (PNG) [file pone.0200272.s009.png]

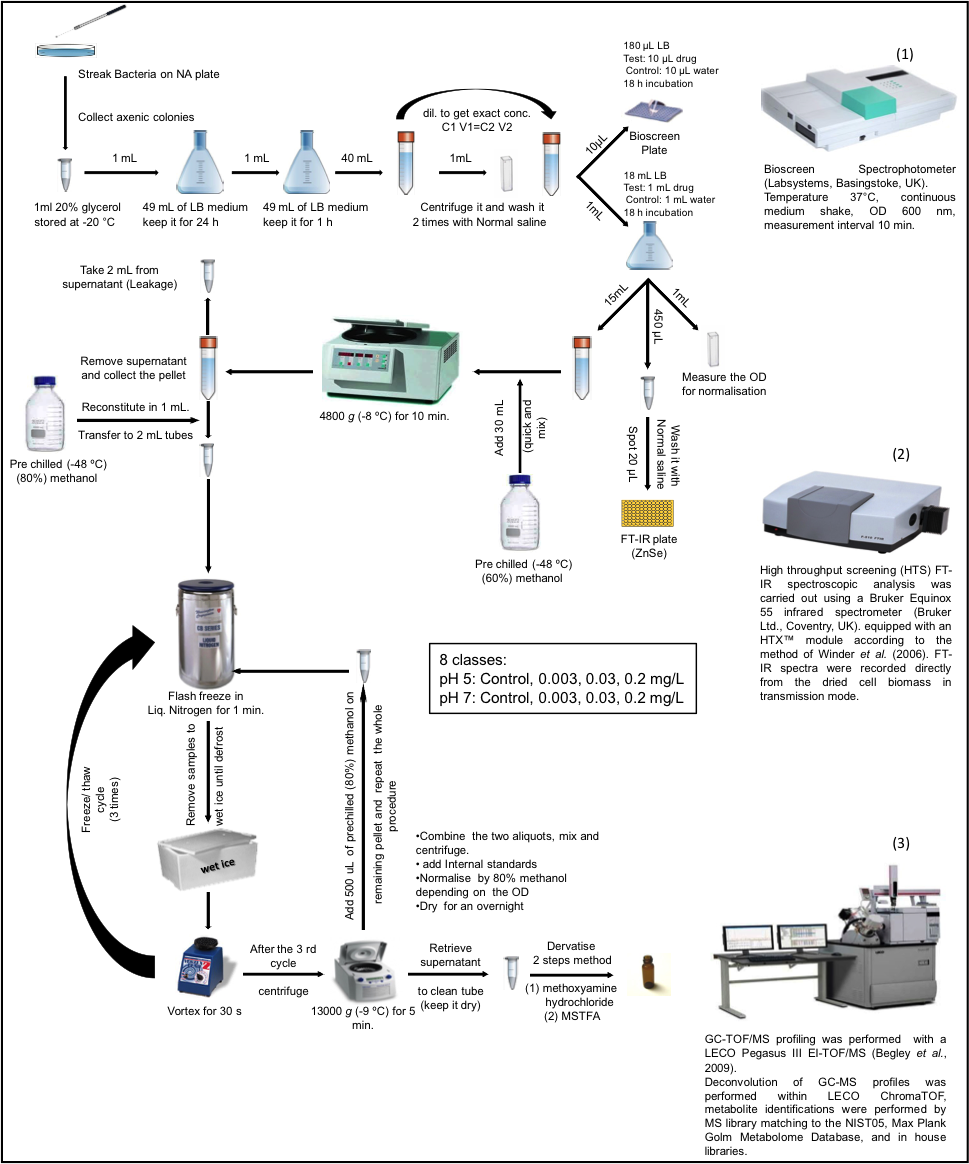

Supplement: S8 Fig — Sample preparation includes: (1) analysis by Bioscreen to determine the MIC of trimethoprim and produce the growth curves of E. coli K-12 at pH 5 and 7 with and without drug challenge. (2) FT-IR analysis of samples after washing with normal saline. (3) GC-MS analysis of samples after quenching and extraction using 60% and 80% cold (-48°C) methanol respectively. (PNG) [file pone.0200272.s010.png]

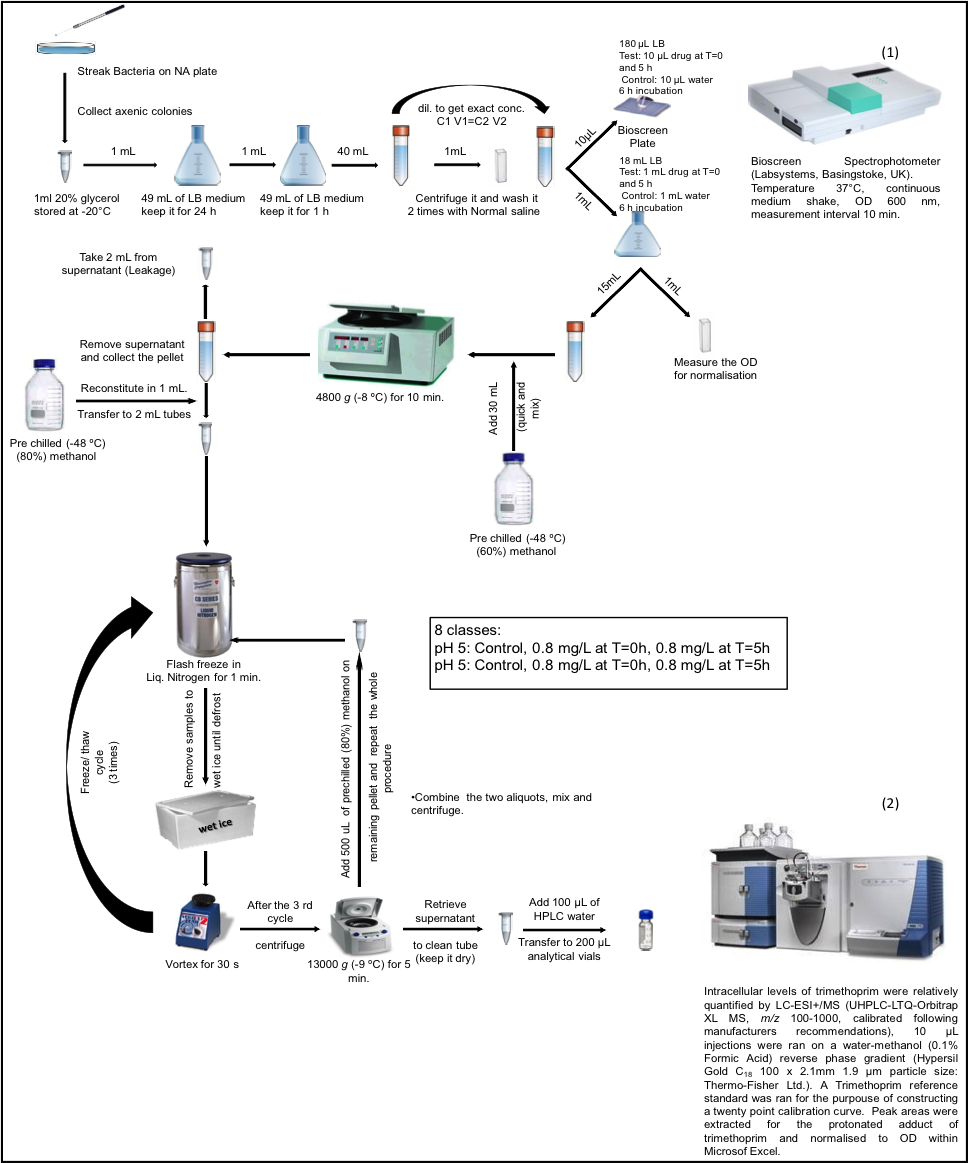

Supplement: S9 Fig — Sample preparation includes: (1) analysis by Bioscreen to produce the growth curves of E. coli K-12 at pH 5 and 7 after challenge with 0.8 mg L-1 of trimethoprim added at two time points: (I) at the beginning of the lag phase (time = 0 h) and (II) at the mid-exponential phase (time = 5 h). (2) LC-MS analysis of sample extracts, for relative quantification of the intracellular drug levels after quenching and extraction using 60% and 80% cold (-48°C) methanol respectively. (PNG) [file pone.0200272.s011.png]
